# The network structure of posttraumatic stress symptoms in war‐affected children and adolescents

**DOI:** 10.1002/jcv2.12124

**Published:** 2022-12-28

**Authors:** Florian Scharpf, Laura Saupe, Anselm Crombach, Roos Haer, Hawkar Ibrahim, Frank Neuner, Kirsi Peltonen, Samir Qouta, Regina Saile, Tobias Hecker

**Affiliations:** ^1^ Department of Psychology Bielefeld University Bielefeld Germany; ^2^ Institute for Interdisciplinary Research on Conflict and Violence Bielefeld University Bielefeld Germany; ^3^ Department of Psychology University Eichstätt‐Ingolstadt Eichstätt Germany; ^4^ Department of Psychology Konstanz University Konstanz Germany; ^5^ Department of Psychology Saarland University Saarbrücken Germany; ^6^ Institute of Political Science Leiden University Leiden Netherlands; ^7^ Research Center for Child Psychiatry University of Turku Turku Finland; ^8^ School of Social Sciences and Humanities Doha Instiute for Graduate Studies Al Daayen Qatar; ^9^ Treatment Center for Victims of Torture Ulm Germany

**Keywords:** adolescents, children, conflict, network analysis, posttraumatic stress disorder, PTSD

## Abstract

**Background:**

It is unclear whether findings from previous network analyses of posttraumatic stress disorder (PTSD) symptoms among children and adolescents are generalizable to youth living in war‐torn settings and whether there are differences in the structure and connectivity of symptoms between children and adolescents. This study examined the network structure of PTSD symptoms in a sample of war‐affected youth and compared the symptom networks of children and adolescents.

**Methods:**

The overall sample comprised 2007 youth (6–18 years old) living in Burundi, Democratic Republic of Congo, Iraq, Palestine, Tanzania, and Uganda amid or close to war and armed conflict. Youth reported their PTSD symptoms using a self‐report questionnaire in Palestine and structured clinical interviews in all other countries. We computed the networks of the overall sample and of two sub‐samples of 412 children (6–12 years) and 473 adolescents (13–18 years) and compared the structure and global connectivity of symptoms among children and adolescents.

**Results:**

In both the overall sample and the sub‐samples, re‐experiencing and avoidance symptoms were most strongly connected. The adolescents' network had a higher global connectivity of symptoms than the children's network. Hyperarousal symptoms and intrusions were more strongly connected among adolescents compared to children.

**Conclusion:**

The findings lend support to a universal concept of PTSD among youth characterized by core deficits in fear processing and emotion regulation. However, different symptoms may be particularly important in different developmental stages, with avoidance and dissociative symptoms dominating in childhood and intrusions and hypervigilance gaining importance in adolescence. Stronger symptom connections may render adolescents more vulnerable to the persistence of symptoms.

## Graphical abstract

The present study examined the network structure of posttraumatic stress symptoms among war‐affected children and adolescents and tested for age differences. Re‐experiencing and avoidance symptoms were most strongly connected in the overall network. The adolescents' network exhibited a higher overall connectivity of symptoms and greater centrality of intrusions and hyperarousal symptoms than the children’s network. The findings point to a universal concept of PTSD in childhood and adolescence and emphasize the importance of considering the developmental stage in the conceptualization, diagnosis and treatment of the disorder.

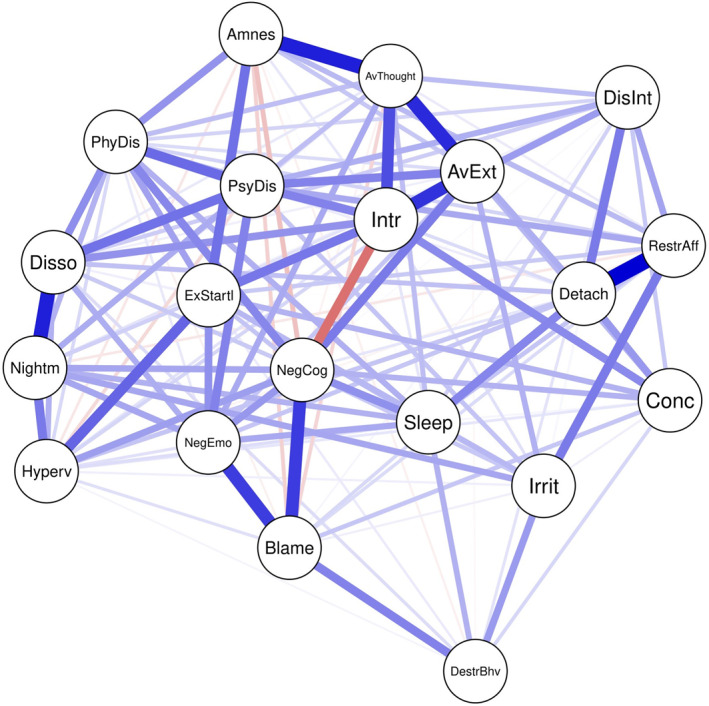

**Key points**

- Re‐experiencing and avoidance symptoms were most strongly connected in the network of war‐affected children and adolescents. Overall connectivity of symptoms was higher among adolescents than among children. Intrusions and hyperarousal symptoms exhibited higher centrality among adolescents. Dissociative reactions were most and hypervigilance was least central for children, whereas intrusions were most central and amnesia was least central for adolescents.
- These findings point to a universal conceptualization of posttraumatic stress disorder (PTSD) with underlying disruptions of fear processing and emotion regulation and suggest a differential expression of PTSD symptoms in childhood and adolescence.
- Age differences in the presentation of PTSD symptoms should be more strongly considered in research and clinical practice

The majority of children and adolescents growing up in areas of armed conflict are exposed to multiple traumatic events, increasing their risk of developing symptoms of posttraumatic stress disorder (PTSD) (Ehntholt & Yule, 2006; Scharpf et al., 2021). PTSD is related to significant immediate and long‐term psychological suffering and impairment, including lower academic achievement and increased risk of suicide attempts, substance abuse and delinquency (Bolton et al., 2004; LeBouthillier et al., 2015; McFarlane, 2010). In the major diagnostic systems, the Diagnostic and Statistical Manual of Mental Disorders Fourth (DSM‐IV) and Fifth Edition (DSM‐5) and the International Classification of Diseases 11th Revision (ICD‐11), symptoms are only assumed to co‐occur because they are caused by a common underlying disorder. Recently, a network systems perspective has gained increasing attention as an alternative conceptualization of psychopathological symptoms (Birkeland et al., 2020). This approach emphasizes the complex associations between symptoms, mutually causing and reinforcing each other (Borsboom & Cramer, 2013). Sufficiently strong between‐symptom associations may generate self‐sustaining feedback loops, which may result in a disorder state (Borsboom, 2017). Within a network, some symptoms may be more influential than others, implying that they are related to a larger number of other symptoms (Weems et al., 2019). Although the clinical utility of findings from network analyses has yet to be established, such central symptoms may represent plausible targets for interventions (Fried et al., 2018).

Few studies have applied this approach to networks of PTSD symptoms among trauma‐exposed children and adolescents. For instance, in a clinical sample in the United States, Germany and Norway, Bartels et al. (2019) identified symptoms related to negative alterations in cognition and mood as most central to the network. De Haan et al. (2020) drew on a large dataset from eight different countries and found symptoms related to the re‐experiencing cluster to be most central to the network. To date, however, evidence on the network of PTSD symptoms among conflict‐affected children and adolescents is scarce, although it is estimated that one in six children worldwide live in areas impacted by armed conflict (Save the Children, 2019). The few existing studies only included conflicted‐affected youth as a subgroup (de Haan et al., 2020) or relied on small samples of predominantly male and unaccompanied youth living in secure high‐income settings (Pfeiffer et al., 2019; Schumacher et al., 2021), limiting the generalizability to other groups of conflict‐affected youth living in active and post‐conflict regions. As the severity and patterns of PTSD symptoms can vary extremely based on the type and number of traumatic events (Alisic et al., 2014; Cloitre et al., 2009; Kelley et al., 2009), it is conceivable that the PTSD symptom networks of conflict‐affected youth may differ from youth who have been mainly exposed to other traumatic events.

Calls have been made for a stronger consideration of developmental factors in the conceptualization, diagnosis and treatment of PTSD among children and adolescents (Carrion et al., 2002; Scheeringa et al., 2011). Most PTSD symptoms are related to disruptions of memory (e.g. intrusions, dissociative reactions), emotions (e.g. blunted affect, loss of interest) and executive function (e.g. irritability, hypervigilance). The main neural substrates underlying these functions, i.e. the hippocampus, the amygdala and the prefrontal cortex, undergo significant structural and functional changes from early childhood up to adulthood (Weems et al., 2019). For instance, while age‐related increases of hippocampal volume and the connectivity between the amygdala and prefrontal cortex as well as a decrease in amygdala reactivity to emotional stimuli appear to underly the improvement of emotion regulation capacities in typically developing youth, the reverse neurodevelopmental pattern may account for heightened threat reactivity and reduced emotion regulation in youth with PTSD over time (Herringa, 2017). Although these age‐related findings may imply phenotypic differences in the presentation of PTSD among children and adolescents, so far only one study examined the role of age in the networks of youth's PTSD symptoms. Russell et al. (2017) observed both differences and similarities in the networks of children (ages 8–13) and adolescents (ages 14–18) exposed to Hurricanes Katrina and Gustav. Although the networks did not differ in the overall strength of connections, certain symptoms, e.g. amnesia and numbness of negative affect, exaggerated startle response and hypervigilance, were more strongly connected among children than among adolescents. In addition, physiological reactivity and avoiding activities emerged as the most central symptoms among children, whereas numbness of positive affect and nightmares were most central among adolescents.

The present study drew on a large international dataset of children and adolescents who were living in or close to regions affected by war and armed conflict. Despite this homogeneity in terms of conflict exposure, the sample is also heterogeneous regarding the youth's ethnic background and the specific conflict setting, thus representing different groups of conflict‐affected youth. In addition, the dataset comprised youth in different developmental stages from middle childhood up to late adolescence. These unique characteristics of the sample allowed us to pursue two objectives: (a) to examine the network structure of PTSD symptoms in a large sample of war‐exposed children and adolescents, thereby expanding the currently scant evidence for this group in the network literature; (b) to compare the symptom networks of children (6–12 years old) and adolescents (13–18 years old), which may yield insights into developmental differences in the presentation and expression of PTSD symptoms.

## METHOD

### Participants

The dataset consisted of six samples from studies conducted in Burundi, Democratic Republic of Congo (DRC), Gaza (Palestine), Iraq, Tanzania and Uganda (detailed descriptions of samples and conflict settings can be found in Appendices S1–S6). It comprised overall 2007 children and adolescents (49.8% girls) aged 6–18 years (M = 11.92, SD = 2.70). Characteristics of and comparisons between the (sub)sample(s) are depicted in Table S1.

### Measures

The Gaza sample used the Children’s Impact of Events Scale 13‐item version (CRIES‐13), which captures nine DSM‐5 symptoms related to re‐experiencing, avoidance and hyperarousal using a 4‐point Likert scale. In the DRC, the International Trauma Questionnaire was used to assess the six PTSD symptoms according to ICD‐11 on a 5‐point Likert scale. In Uganda, the University of California at Los Angeles (UCLA) PTSD Reaction Index for DSM‐IV (UPID) with 22 items rated on a 5‐point Likert scale was used to assess the 17 PTSD symptoms according to DSM‐IV. In Tanzania and Burundi, the 27 items of the UCLA PTSD Reaction Index for DSM‐5 served as a measure of the 20 symptoms of PTSD according to DSM‐5. In Iraq, the Posttraumatic Stress Interview for Children (KID‐PIN; Ibrahim et al., 2021) was applied. The KID‐PIN consists of 20 items that reflect DSM‐5 criteria for PTSD on a 5‐point Likert scale. When individual symptoms were assessed by more than one item, the highest score of these items was used. To increase comparability of measures (Fried et al., 2018), the CRIES‐13 was rescaled to the same range as the other instruments (0–4). The measures were administered in an individual structured clinical interview format in all samples except for the Gaza sample, where measures were administered as questionnaires in the classroom setting.

### Ethical Considerations

The studies had been approved by the respective responsible ethical committees and all children and adolescents had given their informed consent for participation (see Appendices S1–S6 for details).

### Missing data

Overall, there were 14 participants with one missing value each in PTSD symptoms, which were replaced with the participant's mean score in the scale. However, due to the different measures and conceptualizations of PTSD across samples, not all participants had information on every PTSD symptom. For instance, complete information on all 20 DSM‐5 symptoms was available for 905 participants and on the 17 DSM‐IV symptoms for 1273 participants. In line with previous work (de Haan et al., 2020; Fried et al., 2018), we retained all participants in the analysis for the overall sample and estimated correlations among symptoms based on pairwise complete observations (Fried et al., 2018). Since the analyses involving age comparisons required complete information for all participants, we included the participants with complete information on the 17 DSM‐IV symptoms (*n* = 1273) for sub‐networks based on age.

### Data analyses

We estimated the network of the 20 DSM‐5 symptoms of PTSD in the overall sample (*n* = 2007). For age‐specific analysis, we divided the participants with complete data on the 17 DSM‐IV symptoms into two groups: children (ages 6–12; *n* = 800) and adolescents (ages 13–18; *n* = 473). Adolescents had significantly higher levels of PTSD symptoms than children (M_children_ = 6.49, M_adolescents_ = 13.14, *t* = −10.45, *p* < .001). As the differing symptom severity may confound comparisons between networks, we excluded all children with very low symptom levels (< sum score of 3; *n* = 388). The resulting sub‐sample of children (*n* = 412) did not significantly differ from the adolescent sub‐sample in terms of gender composition, trauma load and PTSD symptom severity (see Table S2) and was therefore used for analyses. The density plots for each PTSD symptom indicated that the distributions were largely equivalent across age groups (see Figure S1), suggesting minimal difference in overall score variance (Russell et al., 2017). Descriptive statistics of each symptom in the overall sample and in the two age groups are displayed in Table S3.

We first estimated the networks, computed centrality indices and evaluated network stability using the R packages *qgraph* (Epskamp et al., 2012) and *bootnet* (Epskamp et al., 2018). Specifically, the networks were estimated using a *Graphical Gaussian Model*, in which associations (i.e. edges in network terminology) between symptoms (i.e., nodes) represent estimates of partial correlations. A connection between two symptoms in the resulting network implies that they are dependent after controlling for all other symptoms. Edges were calculated based on polychoric correlations due to the ordinal scale level of PTSD symptoms.

As indicators of the centrality of symptoms, we estimated each symptom's *node strength,* the sum of absolute edge weights (both positive and negative weights) connected to a node, *expected influence*, the sum of a node's edge weights taking into account negative edges, *closeness* (only in the overall network), quantifying how well a node is indirectly connected to other nodes, and *predictability* (only in sub‐networks), representing a node's shared variance with all of its direct neighbors. To test whether differential variability (restricted range) in symptom severity ratings may distort conclusions about symptom importance (Terluin et al., 2016), we computed correlations between the centrality indicators and standard deviation in symptom severity ratings (McNally et al., 2017).

To evaluate the accuracy and stability of each network, we estimated the 95% confidence intervals (CIs) around the edge weights by bootstrapping and calculated the correlation stability coefficients (CS‐coefficient) for the centrality indices (Epskamp et al., 2018). The CS‐coefficient quantifies the maximum proportion of cases that can be dropped to retain a correlation of ≥0.7 between original centrality indices and centrality indices of networks based on subsets with 95% probability. According to Epskamp et al. (2018), CS‐coefficients of at least 0.25 and 0.5 indicate moderate and strong stability of the order of centrality indices respectively. Further, centrality difference tests were conducted to examine differences among nodes in terms of centrality.

Finally, we compared the sub‐networks of children and adolescents along two aspects using the R package *NetworkComparisonTest (NCT)* (Van Borkulo, 2019): (a) network structure invariance, i.e. whether the networks differ in terms of their overall structure; (b) global strength invariance, i.e. whether the networks differ in their overall level of connectivity defined as the sum of the absolute value of all edge weights. Specifically, the NCT applies a permutation‐based algorithm that repeatedly calculates differences in structure and connectivity between sub‐networks by randomly regrouping individuals (1000 times). Two test statistics *M* and *S* reflect the magnitude of differences in network structure and global strength, respectively. In addition, we compared each node's strength and expected influence across sub‐networks. The R script for all analyses can be found in Appendix S7.

## RESULTS

### Overall network

**FIGURE 1:**
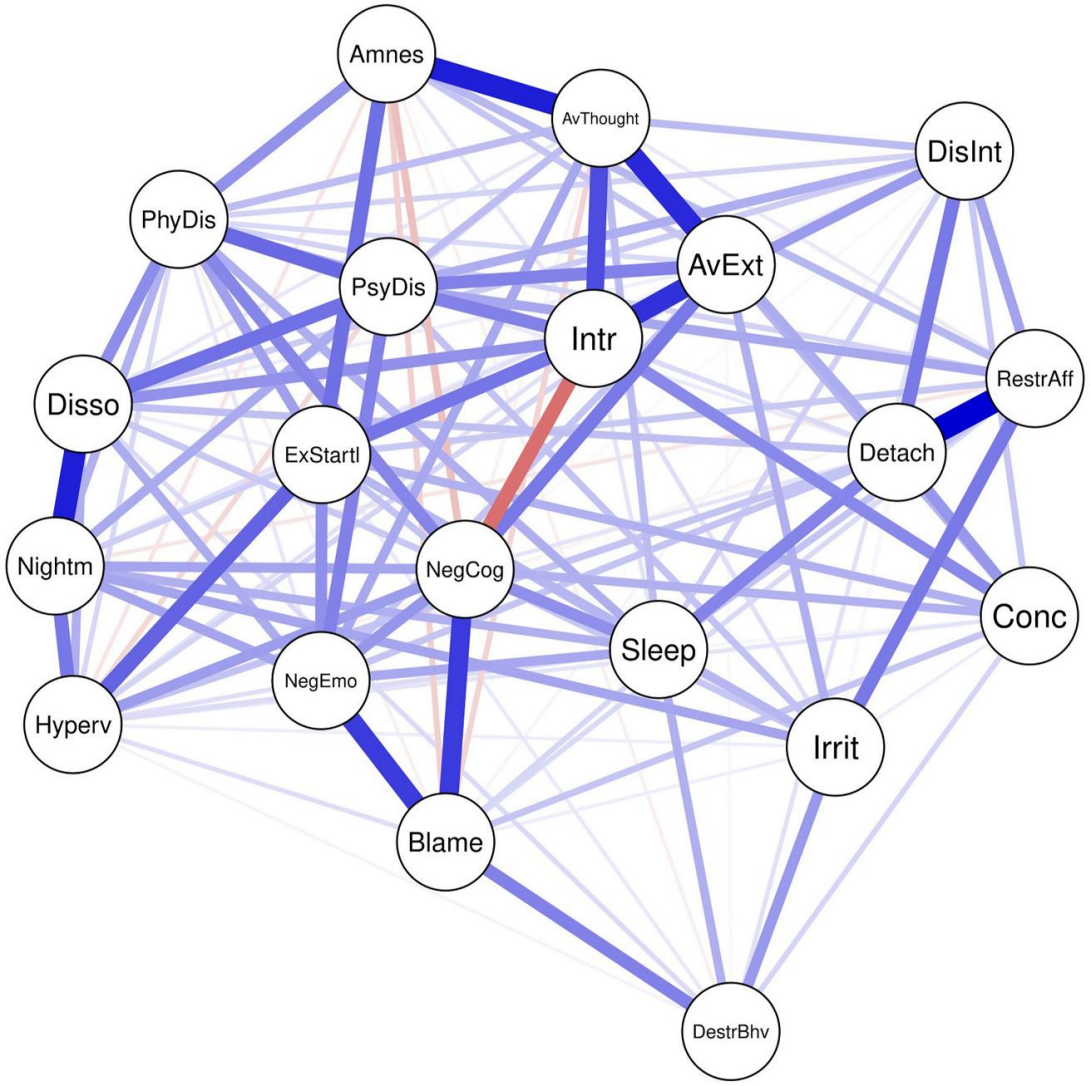
Network of 20 posttraumatic stress disorder symptoms according to Diagnostic and Statistical Manual of Mental Disorder Fifth Edition in the overall sample (*n* = 2007). The edge color indicates the direction of associations (blue for positive and red for negative associations), while thicker and more saturated edges reflect stronger associations. Intr = Intrusions, Nightm = Nightmares, Disso = Dissociative reactions, PsyDis = psychological distress, PhyDis = physiological reactions, AvThought = avoidance internal, AvExt = avoidance external, Amnes = amnesia, NegCog = negative cognitions, Blame = distorted blaming, NegEmo = persistent negative emotions, Disint = diminished interest, Detach = detachment, RestrAff = restricted affect, Irrit = irritable behavior, DestrBhv = self‐destructive behavior, Hyperv = hypervigilance, ExStartl = exaggerated startle, Conc = concentration problems, Sleep = sleep disturbance

The network of the 20 DSM‐5 symptoms in the total sample (*n* = 2007) is displayed in Figure 1. The network consisted of 138/190 (72.6%) non‐zero edges (mean weight: .047), of which 128 (92.8%) were positive and 10 (7.2%) were negative. Strong positive associations emerged, for instance, between *detachment* and *restricted affect*, between *avoidance of internal reminders* and *amnesia* and between *nightmares* and *dissociative reactions,* while the strongest negative association was between *intrusions* and *negative cognitions*. As the bootstrapped CIs around the edge weights were large and most of the CIs overlapped, the order of the edge weights needs to be interpreted with caution (see Figure S2).

**FIGURE 2:**
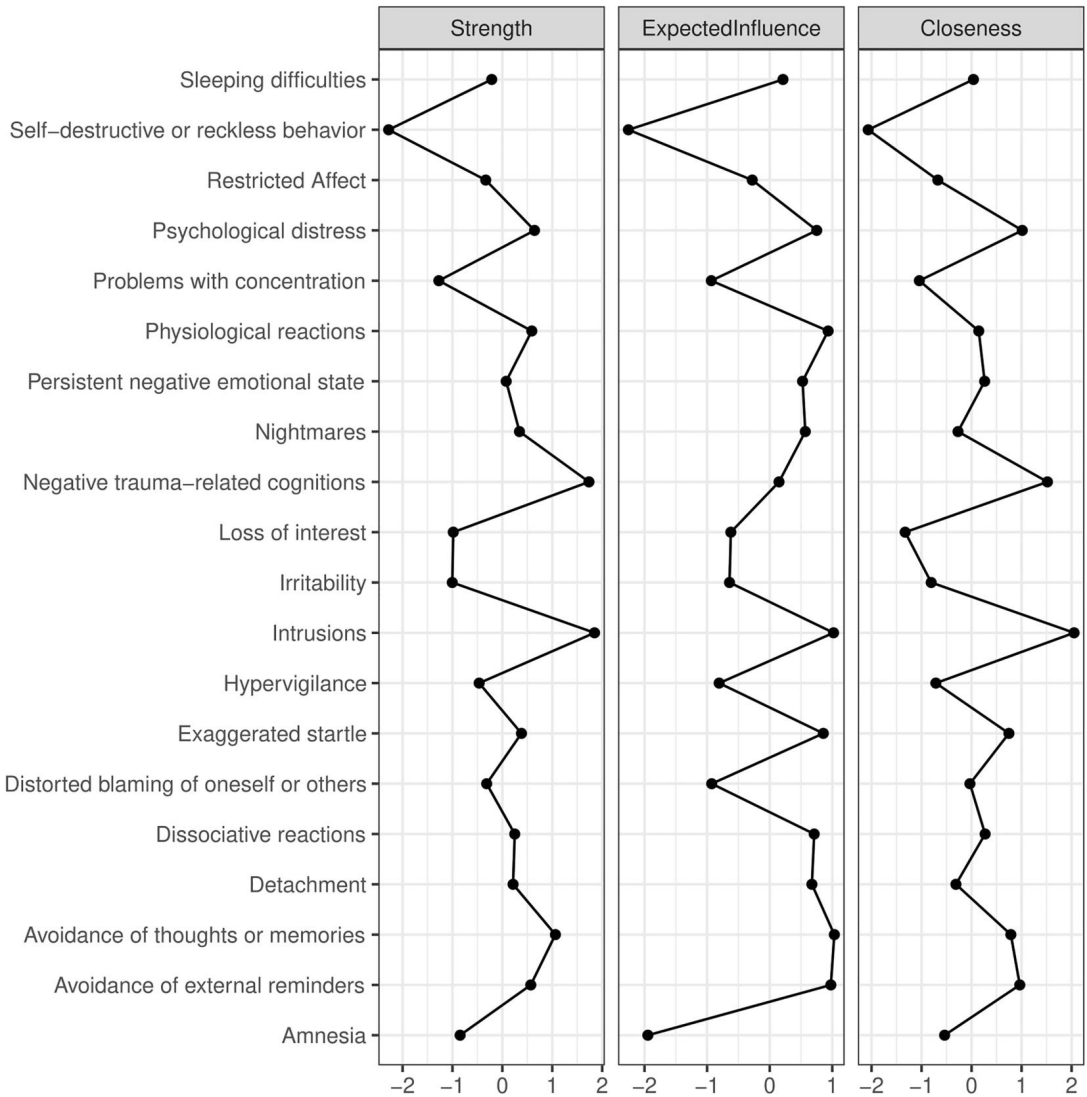
Node strength, expected influence and closeness of 20 posttraumatic stress disorder symptoms according to Diagnostic and Statistical Manual of Mental Disorder Fifth Edition in the overall sample (*n* = 2007)

As shown in Figure 2, *intrusions*, *negative cognitions* and *avoidance of internal reminders* were most central in terms of node strength, *intrusions*, *avoidance of internal reminders*, *avoidance of external reminders*, *physiological reactivity* and *exaggerated startle* were most central in terms of expected influence and *intrusions* and *negative cognitions* were the symptoms with the highest closeness. *Self‐destructive behavior* was least central in all centrality metrics. The most and least central symptoms also significantly differed from most other symptoms in the respective centrality metric (see Tables S3–S5). The CS‐coefficients of 0.67 and 0.75 indicated a strong stability of the order of the node strength and expected influence metric respectively, while the stability of closeness was moderate with a CS‐coefficient of 0.44 (see Figures S6–S8). However, a strong and significant correlation between symptoms' variance and node strength (*r* = 0.65, *p* = .002), expected influence (*r* = 0.73, *p* < .001) and closeness (*r* = 0.68, *p* = .001) indicated that differences in centrality may be due to differences in variability across symptoms.

### Sub‐networks based on age

**FIGURE 3:**
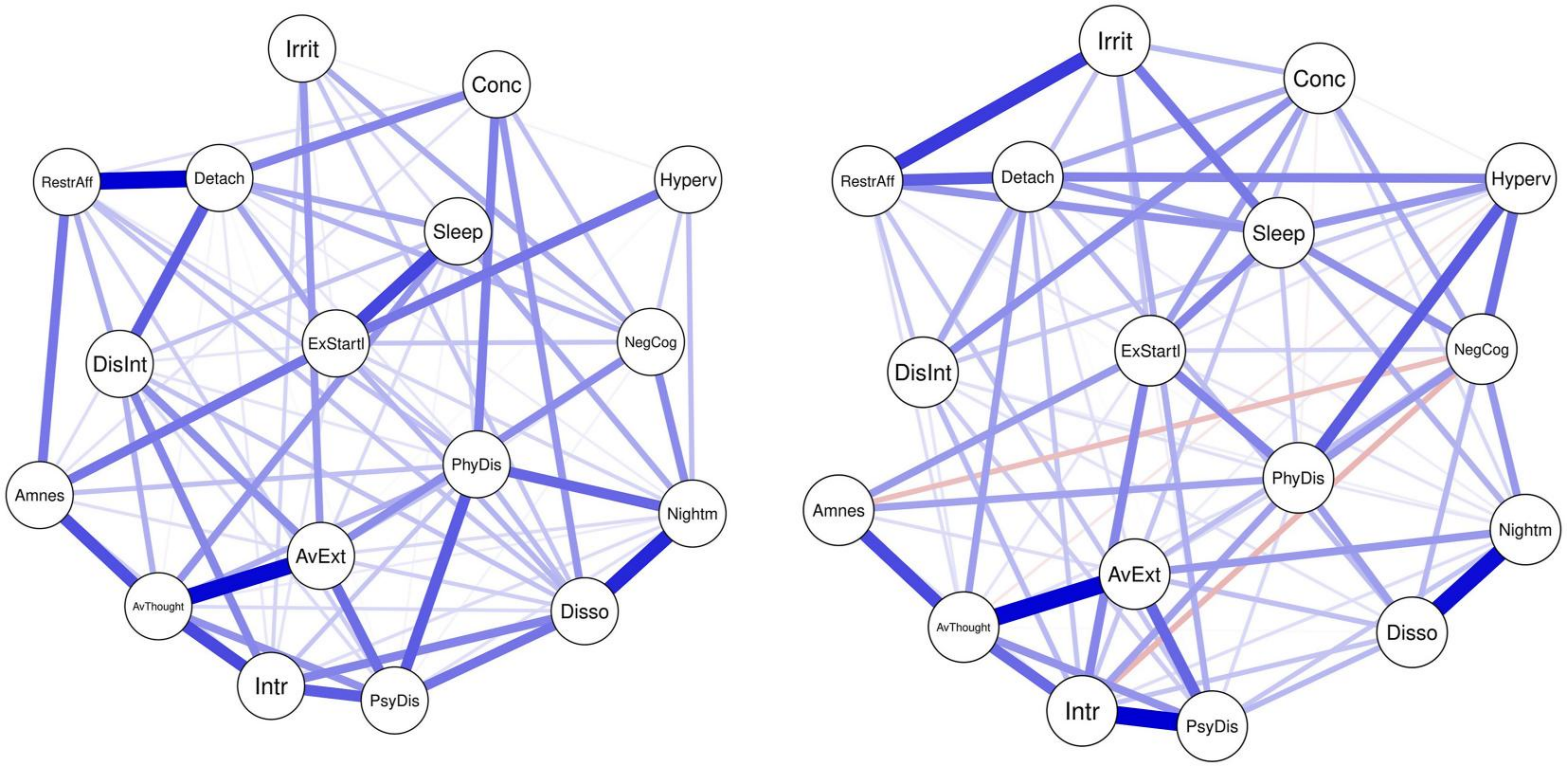
Network of 17 posttraumatic stress disorder symptoms according to Diagnostic and Statistical Manual of Mental Disorder Fourth Edition in children between 6 and 12 years (left, *n* = 412) and adolescents between 13 and 18 years (right, *n* = 473). The edge color indicates the direction of associations (blue for positive and red for negative associations), while thicker and more saturated edges reflect stronger associations. Intr = Intrusions, Nightm = Nightmares, Disso = Dissociative reactions, PsyDis = psychological distress, PhyDis = physiological reactions, AvThought = avoidance internal, AvExt = avoidance external, Amnes = amnesia, NegCog = negative cognitions, Disint = diminished interest, Detach = detachment, RestrAff = restricted affect, Irrit = irritable behavior, Hyperv = hypervigilance, ExStartl = exaggerated startle, Conc = concentration problems, Sleep = sleep disturbance

The networks of the 17 DSM‐IV symptoms in children (ages 6–12; *n* = 412) and adolescents (ages 13–18; *n* = 473) are displayed in Figure 3. The children's network had 86/136 (63.2%) non‐zero edges (mean‐weight: .050), of which all except for one (*intrusions* and *negative cognitions*) were positive. The adolescents' network had 88/136 (64.7%) non‐zero edges (mean‐weight: .056), of which 84 (95.5%) were positive and four (4.5%) were negative. Strong associations in the children's network emerged between *detachment* and *restricted affect*, *avoidance of internal reminders* and *avoidance of external reminders* and between *nightmares* and *dissociative reactions*. Additional strong associations in the adolescents' network were between *intrusions* and *psychological distress*, *restricted affect* and *irritable behavior*, *avoidance of internal reminders* and *amnesia* and *physiological reactivity* and *hypervigilance*. In both sub‐networks, the large and frequently overlapping CIs around edge weights indicate that their order should be interpreted with caution (see Figure S9).

**FIGURE 4:**
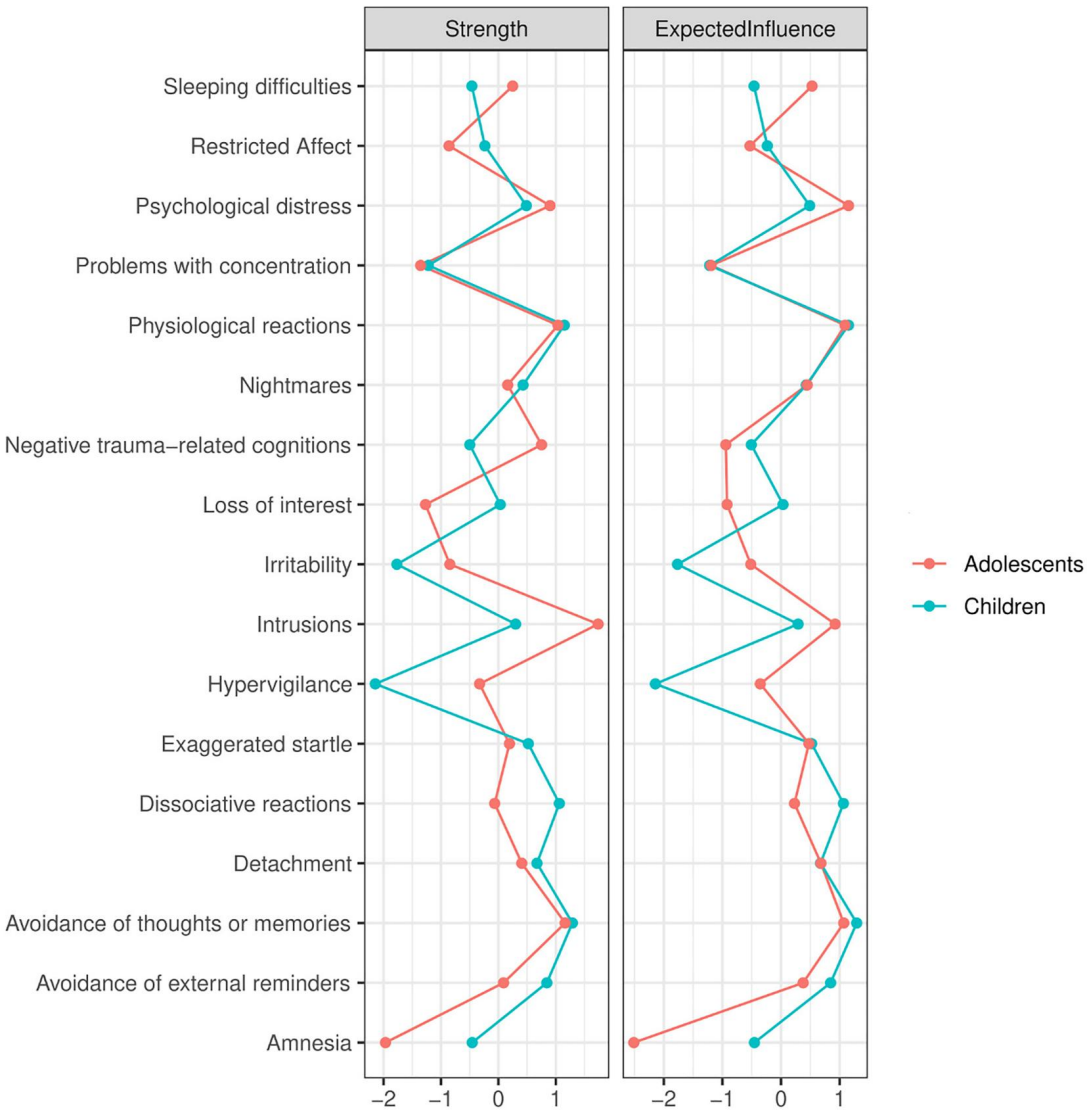
Node strength and expected influence of the 17 posttraumatic stress disorder symptoms according to Diagnostic and Statistical Manual of Mental Disorder Fourth Edition in children (green line) and adolescents (red line)

Among children, *avoidance of internal reminders*, *physiological reactivity*, *dissociative reactions* and *avoidance of external reminders* had the highest centrality in all metrics, while *hypervigilance* had the lowest centrality (see Figure 4 and Table 1). Among adolescents, the most central symptoms in terms of node strength were *intrusions*, *avoidance of internal reminders*, *physiological reactivity*, *psychological distress*, and *negative cognitions*, while *amnesia* was least central. A similar pattern was observed for expected influence and predictability in the adolescents' network, with the exception of a lower centrality of *negative cognitions* in both metrics and a higher centrality of avoidance of external reminders in terms of predictability (see Figure 4 and Table 1). Although the most and least central symptoms significantly differed from most other symptoms in the respective centrality metric, intrusion and avoidance symptoms did not differ from each other (see Figures S10 and S11). The CS‐coefficients indicated a strong stability for node strength (0.52) and expected influence (0.52) among children and a moderate and strong stability for node strength (0.44) and expected influence (0.67) respectively among adolescents (see Figure S12 and Figure S13). Among children, the correlation between symptoms' centrality and their variance was moderate, yet nonsignificant (*r* = 0.38, *p* = .129 for strength and expected influence, *r* = 0.44, *p* = .079 for predictability). Among adolescents, the strong and significant correlation between symptoms' variance and node strength (*r* = 0.65, *p* = .002), expected influence (*r* = 0.77, *p* < .001) and predictability (*r* = 0.87, *p* < .001) suggested that the differential variability across symptoms may account for differences in centrality.

**TABLE 1:** Node strength, expected influence and predictability of symptoms in the sub‐networks based on age

Symptom	Node strength		Expected influence		Predictability	
	Children	Adolescents	Children	Adolescents	Children	Adolescents
Intrusions	0.87^b^	1.20	0.87^d^	1.10	0.43	0.64
Nightmares	0.90	0.97	0.90	0.97	0.37	0.53
Dissociative reactions	1.10	0.94	1.10	0.94	0.46	0.53
Psychological distress	0.91	1.10	0.91	1.10	0.44	0.63
Physiological reactions	1.10	1.10	1.10	1.10	0.46	0.57
Avoidance of internal reminders	1.10	1.10	1.10	1.10	0.53	0.62
Avoidance of external reminders	1.00	0.96	1.00	0.96	0.48	0.58
Amnesia	0.69	0.62	0.69^d^	0.45	0.30	0.29
Negative cognitions	0.68	1.10	0.68	0.73	0.28	0.46
Diminished interest	0.81	0.73	0.81	0.73	0.35	0.36
Detachment	0.96	1.00	0.96	1.00	0.39	0.51
Restricted positive affect	0.74	0.80	0.74	0.80	0.32	0.44
Irritable/aggressive behavior	0.38^a^	0.80	0.38^a^	0.80	0.13	0.43
Hypervigilance	0.29^a^	0.89	0.29^a^	0.83	0.09	0.46
Exaggerated startle	0.92	0.98	0.92	0.98	0.34	0.50
Concentration problems	0.51^d^	0.72	0.51	0.68	0.22	0.35
Sleeping difficulties	0.69^c^	0.99	0.69^c^	0.99	0.29	0.51

^a^ *p* < .001.

^b^ *p* < .01.

^c^ *p* < .05.

^d^ *p* < .10.

Children and adolescents' network did not significantly differ in overall network structure (test statistic M = 0.24, *p* = .055). However, the adolescents' network had a significantly higher global strength than the children's network (8.04 vs. 6.79; test statistic *S* = 1.26; *p* < .001). As shown in Table 1, *hypervigilance*, *irritable behavior* and *sleep disturbance* had a significantly higher node strength and expected influence in the adolescents' network compared to the children's network, while *intrusions* had a significantly higher node strength, but not expected influence, among adolescents than among children. The predictability metric revealed that out of all symptoms the two avoidance symptoms shared most variance with their direct neighbors in the children's network, followed by *dissociative reactions* and *physiological reactions*. *Hypervigilance* was predicted least well by its neighbors in the children's network. In the adolescents' network, however, *intrusions*, *psychological distress* and *avoidance of internal reminders* were the symptoms that were explained best by their neighbors, whereas *amnesia* shared least variance with its neighbors.

## DISCUSSION

The present study is the first to examine the network structure of PTSD symptoms among children and adolescents living in conflict‐affected areas and yields further insights into developmental differences in symptom presentation.

It is striking that the symptom network in our sample exhibited many similarities with the symptom networks observed among youth with different cultural backgrounds, living contexts and trauma exposures. These similarities include the strong associations between nightmares and dissociative reactions (Cao et al., 2019), between avoidance of internal and of external reminders (Bartels et al., 2019) and between restricted affect and detachment (Bartels et al., 2019; Russell et al., 2017). Re‐experiencing symptoms, particularly intrusions, psychological distress and physiological reactions, emerged as the most strongly connected symptoms in the overall network, which was a consistent pattern across different centrality indicators. While these symptoms have also been identified as central in previous network analyses (Bartels et al., 2019; Cao et al., 2019; de Haan et al., 2020; Russell et al., 2017), it makes sense that re‐experiencing and avoidance symptoms are central for youth living in or close to areas of ongoing conflict, where reminders of traumatic experiences may be omnipresent. In our view, the fact that we could replicate previous findings on the network structure of PTSD symptoms among youth from mainly Western settings in our large ethnically diverse sample of conflict‐affected youth from East African and Middle Eastern backgrounds provides strong support for a universal conceptualization of PTSD in childhood and adolescence. From this perspective, pediatric and adolescent PTSD might be best characterized by emerging and gradually deteriorating disruptions of core cognitive and affective functions such as fear processing and emotion regulation and their neural substrates, particularly the hippocampus and amygdala (Herringa, 2017; Liberzon & Abelson, 2016; Weems et al., 2019). These disruptions then give rise to the phenotype of PTSD in youth, which appears to manifest itself predominantly in the form of re‐experiencing and avoidance symptoms. It is important to note that in such a universal perspective the cultural context still has considerable influence on the expression of symptoms and on their underlying psychological processes (Liddell & Jobson, 2016).

Intriguingly, the comparison of children and adolescents' symptom networks suggests that the presentation of PTSD depends on the developmental stage. We observed a greater overall connectivity between the 17 DSM‐IV symptoms in the adolescent network compared to the child network. Given the lack of significant differences between age groups in potentially important confounding factors, e.g. gender composition (Cao et al., 2019), trauma load (Kelley et al., 2009) and symptom severity (Van Borkulo et al., 2015), this may reflect stronger feedback loops between symptoms and thus a greater vulnerability to the persistence of symptoms in adolescents. We also observed age differences in terms of the centrality of individual symptoms. While avoidance of internal reminders and physiological reactivity were central in both groups, intrusions and psychological distress were the most influential re‐experiencing symptoms for adolescents and dissociative reactions for children. The least central symptoms also differed for both age groups, namely hypervigilance for children and amnesia for adolescents. Intrusions and the three hyperarousal symptoms hypervigilance, irritable behavior and sleep disturbance were more central among adolescents than among children.

Considering that the typical development of the neural substrates underlying the key functions that are likely to be disrupted in PTSD is marked by significant changes across childhood and adolescence (Fuhrmann et al., 2015; Tottenham & Gabard‐Durnam, 2017), it is likely that the observed differences in symptom networks reflect the impact of traumatic stress on neurodevelopment in different development stages. While declining amygdala reactivity and increasing connectivity with prefrontal regulatory brain regions appear to be responsible for the typically observed age‐related improvements in regulating negative emotions (Martin & Ochsner, 2016), studies suggest the opposite pattern for youth with PTSD, i.e. age‐related increases of amygdala reactivity and reduced coupling with prefrontal areas, which may lead to higher threat reactivity and weaker emotion regulation in adolescence (Herringa, 2017). This may explain our finding of higher importance of intrusions, psychological distress, hypervigilance, and irritable behavior among adolescents. On the contrary, preliminary findings suggest that children with PTSD may actually show a compensatory engagement of prefrontal areas and downregulation of the amygdala, which is reflected by a lower amygdala activation and higher amygdala‐ventromedial prefrontal cortex coupling compared to typically developing agemates (Herringa, 2017). Given that a neural pattern of dampened amygdala reactivity and heightened amygdala‐prefrontal coupling has also been implicated in dissociative PTSD (Lanius et al., 2010), this may account for the relatively high centrality of dissociative reactions among children.

So far, only one other study (Russell et al., 2017) has compared PTSD symptom networks of children and adolescents. Although that study did not find a difference in the global connectivity of the networks of US‐American children and adolescents who had been exposed to natural disasters, some of our findings were also consistent with those of Russell et al. (2017) and thus corroborate preliminary evidence for developmental differences. For instance, some associations that were observed among children were absent (*amnesia* and *restricted affect*) or markedly weakened (*exaggerated startle* and *hypervigilance*, *diminished interest* and *detachment*) among adolescents. On the other hand, we found more and stronger connections between hyperarousal symptoms and various symptoms from other clusters among adolescents compared to children. These include the links between *irritable behavior* and *restricted affect* and between *hypervigilance* and *physiological reactivity*, *negative cognitions*, or *detachment*. This pattern may reflect deficits in developing executive functions and reduced connections with brain areas related to emotion regulation among older youth (McNally et al., 2015).

It is noteworthy that neuroimaging studies with adults with PTSD have revealed abnormalities in brain networks related to executive function (Akiki et al., 2018). Furthermore, network analyses have consistently identified intrusions and psychological distress as most central and amnesia as least central symptoms among adults (Birkeland et al., 2020). Therefore, our findings point to the intriguing possibility that the symptom network of adolescents reflects the phenotype of emerging neurodevelopmental disruptions that are characteristic of adult PTSD. There may thus be the need for a clearer distinction between pediatric and adult PTSD, with adolescent PTSD representing a transitional form that may show characteristics of both.

### Implications for research and practice

Our findings may have important implications for the conceptualization and treatment of PTSD in childhood and adolescence. Consistent with previous network analyses among youth mainly from Western high‐income settings, symptoms related to re‐experiencing and avoiding traumatic experiences were most strongly connected in our ethnically diverse sample of youth living in non‐Western conflict settings. This lends support to a universal concept of PTSD among children and adolescents, which appears to be characterized by basic disruptions of fear processing and emotion regulation across cultural contexts. This also aligns with the conceptualization of PTSD in the ICD‐11. Furthermore, our findings point to a developmental perspective toward PTSD. Currently, the DSM‐5 applies the same diagnostic criteria to children above the age of six, adolescents and adults, requiring a reduced set of symptoms only for preschool children. However, the observed age differences in local and global connectivity of symptoms suggest that age‐specific symptom manifestations may need to be considered in the diagnosis of PTSD. For instance, as neurodevelopmental effects of trauma on executive brain networks may express themselves as hyperarousal symptoms only in late adolescence, they might not be required for children. Dissociative reactions and avoidance symptoms may represent particularly viable targets in interventions with children, whereas intrusions and emerging deficits in emotion and threat regulation may be promising targets among adolescents. To the extent that the observed age‐related findings truly represent universal neurodevelopmental alterations, they are not limited to this specific sample and generate hypotheses about age differences in the brain structures and functions underlying phenotypic symptom representations, which may be tested in neuroimaging studies.

### Limitations

Several limitations must be noted. First, the cross‐sectional nature of our study precludes assumptions about causality. Longitudinal network analyses are needed to examine true developmental changes in PTSD symptom network structure and connectivity over time. Second, the metrics indicating a higher or lower centrality of certain symptoms need to be interpreted with caution. On the one hand, the different centrality indicators were strongly related to the symptom variance, suggesting that symptoms with greater variance tended to be more central in the network. On the other hand, as discussed in detail by Fried et al. (2018), symptom centrality in itself should be interpreted with care as it does not automatically imply greater clinical relevance of symptoms. Third, possible differences in symptom networks between sub‐samples may have been masked by our analysis. Fourth, we did not examine the role of trauma type and gender in symptom networks, which are likely to interact with age. Similarly, we did not consider comorbid symptoms of PTSD such as depression and anxiety. Fifth, the conceptualization of PTSD symptoms according to DSM‐IV in the age‐specific analyses implied that we could not consider all DSM‐5 symptoms.

## CONCLUSIONS

The network structure and centrality of PTSD symptoms in a large sample of children and adolescents from conflict‐affected regions in East Africa and the Middle East showed many similarities with previous network analyses among youth from Western backgrounds and different trauma exposures. In particular, symptoms related to re‐experiencing and avoidance were most strongly connected in the network. These findings are consistent with the view that traumatic stress has an universal impact on youth's brain functioning, which does not depend on the specific culture or trauma context, and thus lend support to a universal conceptualization of PTSD based on disruptions of fear processing and emotion regulation. In the adolescents' network, the overall connectivity of symptoms was higher and some symptoms, especially related to intrusions and hyperarousal, were more central than in the children's network. These findings may point to a differential impact of traumatic stress on neurodevelopmental structures and functions, which could imply that different symptoms may be most relevant in different developmental stages. Future network studies using longitudinal and experimental designs and combining neurobiological and behavioral markers of functioning are important to understand the development and persistence of trauma‐related psychopathology throughout childhood and adolescence and to identify early targets for prevention and intervention.

## AUTHOR CONTRIBUTIONS

**Florian Scharpf:** Conceptualization, Data curation, Formal analysis, Methodology, Project administration, Software, Validation, Visualization, Writing – original draft, Writing – review & editing. **Laura Saupe:** Data curation, Formal analysis, Investigation, Methodology, Project administration, Visualization, Writing – original draft, Writing – review & editing. **Anselm Crombach:** Data curation, Funding acquisition, Investigation, Writing – review & editing. **Roos Haer:** Data curation, Funding acquisition, Investigation, Writing – review & editing. **Hawkar Ibrahim:** Data curation, Funding acquisition, Investigation, Writing – review & editing. **Frank Neuner:** Funding acquisition, Investigation, Writing – review & editing. **Kirsi Peltonen:** Data curation, Funding acquisition, Investigation, Writing – review & editing. **Samir Qouta:** Funding acquisition, Investigation, Writing – review & editing. **Regina Saile:** Funding acquisition, Investigation, Writing – review & editing. **Tobias Hecker:** Conceptualization, Funding acquisition, Investigation, Methodology, Project administration, Resources, Software, Supervision, Writing – original draft, Writing – review & editing.

## CONFLICTS OF INTEREST

The authors have declared that they have no competing or potential conflicts of interest.

## Supporting information

Supplementary Material

## Data Availability

The data that support the findings of this study are available from the corresponding author upon reasonable request.
