# Supplementary material for: The network structure of posttraumatic stress symptoms in war‐affected children and adolescents
Source: JCPP Adv. 2022 Dec 28;3(1):e12124. doi: 10.1002/jcv2.12124 (PMC10241473; doi:10.1002/jcv2.12124)
Supplement: Supplementary file 1 — Supplementary Material [file JCV2-3-e12124-s001.docx]

Appendix S1: Description of sample from Burundi

**Background and context:** In the aftermath of a 13-year-long civil war insecurities and violence determine the lives of many Burundian people (Amnesty International, 2018, p.107, 108; Uvin, 2009). The small and densely populated country is amongst the poorest countries in the world (Central Intelligence Agency, 2018). In a study regarding childhood maltreatment, all participating children had experienced at least one type of abuse and/or neglect (Charak, de Jong, Berckmoes, Ndayisaba, & Reis, 2017). Especially the rates of childhood EA (32 %) and emotional neglect (93.5 %) were very high. About 14.7 % reported sexual abuse. The high number of sexual violence is also reflected in a report form Psychologues sans Frontiéres Burundi regarding survivors of sexual violence asking for assistance in primary care facilties: In the years of 2011 to 2015, 44 to 57 % of survivors seeking assistance within 72 h after the assault were 13 year or younger (Bambonyé & Crombach, 2017). Probable risk factors for the high number of adverse childhood experiences in Burundi are poverty, lack of child welfare services, poor access to health and education services and residing in conflict zones.

The subsample of Burundian children and adolescents was part of two longitudinal projects regarding the reintegration of children living in the streets of Bujumbura, and of children living on landfills in their families and within the educational system, and a cross-sectional study regarding the impact of sexual assault on mental health symptoms and physical pain. The two longitudinal projects aimed at assessing factors driving children away from their homes and evaluating an intervention program. Most of the children and their families were enrolled in reintegration programs – independently of their participation within the interviews. In 2018 and 2019 some children and their families were enrolled as part of a control group.The longitudinal projects were implemented with the help of local Non-Governmental Organizations providing assistance to these vulnerable populations. In the cross-sectional study on sexual assault, recruitment was conducted in collaboration with a primary care facility in Burundi, which invited their clients to participate in the study.

**Data collection procedures:** In the longitudinal projects, 259 children and adolescents were assessed over a period of four years. The initial assessments, which have been used in the present study, were conducted in spring of each year. The subsample of the cross-sectional study regarding the impact of sexual assault consisted of 143 children and adolescents who were assessed in 2017. In all projects, children and adolescents were selected using convenience sampling and were interviewed by local psychologists who had received intensive training and were supervised weekly by international experts.

**Measures:** All questionnaires have been used in previous projects with vulnerable populations. The measures had been translated to Kirundi, the national language in Burundi, using blind-back translation The children and adolescents were asked about traumatic and violent experiences, their current mental health symptoms, and their social relationships.

**Consent and ethics:** The study received ethical approval by the Université Lumière de Bujumbura, Burundi. Before each interview, the content, procedure, risks, the right to withdraw, and confidentiality was explained, and informed consent was obtained from the children and their caretakers. In case, caretakers were not available, legal advisors of the NGOs working with these children were present as guardians. Participants received a small reimbursement for their time (approximately 2.5 – 5 US dollars depending on the specific project). In case of acute need for assistance, help was provided by trained psychologists and the primary care facility.

**References:**

Amnesty International (2018). *Amnesty International Report 2017/18*. London, United Kingdom: Amnesty International Ltd.

Bambonyé, M. & Crombach, A. (2017). Étude rétrospective clinique des enfants survivants de viols âgés de 13 ans et moins traités au Centre Seruka à Bujumbura, au Burundi, parmi des agressions sexuelles commises de 2011 à 2015. Département fédéral des affaires étrangères (DFAE), Direction du développement et de la coopération (DDC), PSF-BDI Siège, Bujumbura, République du Burundi.

Central Intelligence Agency. (2018, July). *The world fact book.* Retrieved 10.09, 2019, from https://www.cia.gov/library/publications/the-world-factbook/geos/by.html

Charak, R., de Jong, J. T. V. M., Berckmoes, L. H., Ndayisaba, H., & Reis, R. (2017). Assessing the factor structure of the Childhood Trauma Questionnaire, and cumulative effect of abuse and neglect on mental health among adolescents in conflict-affected Burundi. *Child abuse & neglect*, *72*, 383-392.

Uvin, P. (2013). *Life after violence: A people's story of Burundi*. (1st ed. Aufl.). London: Zed Books.

Appendix S2: Description of sample from DRC Congo

**Background and context:** The subsample of the Democratic Republic of the Congo (DR Congo) is part of a cross-sectional study investigating the effect of war exposure on children. More specifically, it looks at how it affects social relationships with other children, family members, and teachers. The DR Congo is facing one of the world's worst humanitarian disasters, and children are paying the heaviest price. World Vision (2017) has called this disaster ‘one of the worst child protection crises” in the world. As of March 2020, according to the UN, the Democratic Republic of the Congo was home to the largest population of internally displaced persons in Africa, with 5.5 million people displaced, 3.2 million of whom were children. Access to these children in need was seriously hampered in areas where armed groups were active and military operations were ongoing (UN 2020). In addition, an estimated **3 million children are acutely malnourished** (World Food Programme 2021). Schools and hospitals are under attack or destroyed. Human rights violations, such as sexual violence, are widespread and continue to occur. Many military operations have an especially adverse effect on children (UN 2020). Children bear also the grunt of ethnic strife in certain regions. Intercommunal clashes account for 19 percent of all child casualties (UN 2020). One of the most disturbing features of the conflict in the DR Congo has been the widespread and systematic use of children as soldiers. Although reliable estimates are lacking, Congo is one of the countries in which children are used as soldiers on a very large scale. Although an action plan was signed between the government and the UN to end the recruitment of children in 2012, reports still appear that show that recruitment of children by all sides has not stopped (UN 2020).

**Data collection procedures:** To examine how children’s exposure to armed conflict might affect their social relations, information was collected from Congolese children in the period 2018-2019 in Bukavu, the capital of the South Kivu province. With the help and consent of six local child protective organizations,^[[1]](#footnote-1)^ a convenience sample of 315 children, adolescents and young adults were recruited. Participating youths were interviewed by an experienced team, primarily composed out of psychologists, with the help of local translators.

**Measures:** Children were asked about their experiences with the war and several questions concerning their current social relations using standardized measures that had been used with conflict-affected populations in the Great Lakes region before. All questions were discussed with the child protective organizations and pre-tested. **Consent and ethics:** The study received approval by the Ethical Committee of Bielefeld University (No. 2018–202). Before each interview, the content, procedure, risks, the right to withdraw, and confidentiality was explained, and informed consent was obtained from the children and whenever necessary from their supervisors or caretakers. Each participant received a small reimbursement (approximately 5 US dollars), independently on whether they answered the questions or not. A licensed psychologist was always present during data collection to provide psychological support to participants and a referral system with collaborating organizations and a nearby hospital was established.

**References**

World Food Programme. 2021. Democratic Republic of the Congo emergency. Available online via: <https://www.wfp.org/emergencies/kasai-emergency> (Accessed 24.10.2021).

UN. 2020. Children and armed conflict in the Democratic Republic of the Congo Report of the Secretary-General. UN S/2020/1030. Available online via: <https://www.securitycouncilreport.org/un-documents/document/s-2020-1030.php> (Accessed 24.10.2021).

World Vision. 2017. Conflict in Democratic Republic of Congo ‘one of the worst child protection crises’. Available online via: <https://www.wvi.org/it-takes-world/pressrelease/conflict-democratic-republic-congo-%E2%80%98one-worst-child-protection-crises%E2%80%99> (Accessed 24.10.2021).

Appendix S3: Description of sample from Gaza

**Background and context:** The context of the present study is the long-lasting Israeli–Palestinian conflict, where children experienced the war on Gaza 2008/2009 (“Operation Cast Lead” in the Israeli military terms). The 23-day war claimed 1417 Palestinian lives, including 313 children, and injured 5303, including 1606 children. Approximately 100,000 people were displaced (United Nations Human Rights Council, 2009; United Nations Office for the Coordination of Humanitarian Affairs [UN: OCHA], 2009).

**Data collection procedures:** The sampling was conducted in two regions (North Gaza and Gaza City) of the Gaza strip that were the most severely bombed and shelled during the 2008–2009 war on Gaza. In both regions, from a list of schools provided by the Ministry of Education, two schools were randomly selected. In each of the schools, two girls’ and two boys’ fifth and sixth grade level classes were randomly sampled (a total of 16 classes). The planned eligible sample was 500, but, for various logistical reasons, 18 children did not participate. The participants were 482 Palestinian boys (50.6%) and girls (49.4%) who were 10–13 years old (M=11.29, SD=.68). They represent the baseline group of a randomised controlled study of the effectiveness of a psychosocial intervention among war-affected children. Data were collected using self-report questionnaires that were administered to participating children in their classrooms by six trained research assistants who explained all procedures and assisted in case of questions.

**Measures:** The measures used in the study focused on children`s exposure to war trauma, various mental health problems and psychosocial well-being. The measures had demonstrated good psychometric properties in previous studies with war-affected youths in Gaza. All questionnaires were either available in Arabic or translated from English to Arabic using blind-back translation.

**Consent and ethics:** The Ministry of Education and the Gaza Community Mental Health Programme reviewed and ethically approved the study protocol and measures. The headmasters of the schools endorsed the study procedure. Parents received an information sheet that included an explanation of the purpose of the study and a request of parents’ consent for the targeted child to participate in the study. Verbal consent for child’s participation was obtained from parents and children. Children received a small gift for their participation. All children in need of psychological support were provided help in the context of the study.

**Reference**

Diab, M., Peltonen, K., Qouta, S.R., Palosaari, E., & Punamäki, R. (2019). Can functional emotion regulation protect children's mental health from war trauma? A Palestinian study. International Journal of Psychology, 54, 42–52.

Appendix S4: Description of sample from Iraq

**Background and context:** The Iraqi subsample contains samples of Iraqi and Syrian children and youth who had fled to the refugee settlements in the north of Iraq due to the war and political conflicts that took place in the Middle East after the Arab Spring events. Arab Spring was a series of peaceful pro-democracy protests and uprisings in the Arab World turned into large-scale civil and international wars that devastated many affected countries in the Arab world, including Iraq and Syria. According to the United Nations High Commissioner for Refugees (UNHCR) data, since began Arab Spring, more than half of Syria's total population (13.5 million) as many as two million Iraqis had been forced to flee their homes and seek safety either inside or outside of their countries (United Nations High Commissioner for Refugees, 2019, 2021).

North of Iraq (or Kurdistan region of Iraq), which is an autonomous region in Iraq, is home to millions of Iraqi and Syrian displaced people. Recent scientific studies (Goessmann et al., 2020; Ibrahim et al., 2019; Mahmood et al., 2019) on Iraqi and Syrian adult refugees residing in the north of Iraq has documented high levels of direct experiences of the war-related traumatic events with high prevalence rates of psychopathological symptoms.

**Data collection procedures:** The Iraq subsample data were collected between March and April 2019 in the Arbat camps in Sulaymaniyah Governorate of the Kurdistan Region of Iraq. A random selection of households was chosen using a spin-the-pen method to determine a direction and a computer-generated sequence of random numbers to select houses in the directions. One child per household was randomly chosen to participate. Fifteen trained paraprofessionals (psychologists and social workers) conducted semi-structured interviews with 174 Iraqi and 158 Syrian displaced children and youths aged between 8 and 16 years old (M=12.67, SD= 2.08). 172 (51.8%) of participants were female and they had completed between 0–10 years of formal educations (M= 4.96, SD= 2.16). Iraqi and Syrian children and youth were exposed to 0 and 20 traumatic event types (M = 4.45, SD = 3.83). A detailed methodological procedure of sampling, data management, and ethical considerations are described elsewhere (Ibrahim et al., 2021).

**Measures:** Children were interviewed about their exposure to war-related trauma and to family violence, various mental health problems and parenting styles using standardized measures, which had been either adapated to or developed for the specific study context. The former were translated from English to Kurdish and Arabic using blind-back translation, while the latter were developed in the languages of the participants.

**Consent and ethics:** Before the interview, the local interviews explained the study and its aims to the parents and the participating child. Both participants provided verbal informed consent for their child`s participation. Both ethics committees of Bielefeld University in Germany (reference number: EUB 2015-046) and Koya University in Iraq (reference number: SHETC-1), as well as local government departments in the north of Iraq such as Directorate of Social Affairs in the Ministry of Labour and Social Affairs and the Joint Crisis Coordination Centre in the Ministry of Interior, has approved the study and its procedure. Neither participating children nor their parents received any compensation for their participation. A protection and referral system was established for those participants who were in need of psychological support.

**References**

Goessmann, K., Ibrahim, H., & Neuner, F. (2020). Association of War-Related and Gender-Based Violence With Mental Health States of Yazidi Women. *JAMA Network Open*, *3*(9), e2013418. https://doi.org/10.1001/jamanetworkopen.2020.13418

Ibrahim, H., Catani, C., Ismail, A. A., & Neuner, F. (2019). Dimensional Structure and Cultural Invariance of DSM V Post-traumatic Stress Disorder Among Iraqi and Syrian Displaced People. *Frontiers in Psychology*, *10*(JULY), 1505. https://doi.org/10.3389/fpsyg.2019.01505

Ibrahim, H., Catani, C., & Neuner, F. (2021). The posttraumatic stress interview for children (KID-PIN): development and validation of a semi-structured interview of PTSD symptoms among displaced children in the Middle East. *PeerJ*, *9*, e12403. https://doi.org/10.7717/PEERJ.12403

Mahmood, H. N., Ibrahim, H., Goessmann, K., Ismail, A. A., & Neuner, F. (2019). Post-traumatic stress disorder and depression among Syrian refugees residing in the Kurdistan region of Iraq. *Conflict and Health*, *13*(1). https://doi.org/10.1186/s13031-019-0238-5

United Nations High Commissioner for Refugees. (2019). *UNHCR - UNHCR Global Trends 2018*. https://www.unhcr.org/statistics/unhcrstats/5d08d7ee7/unhcr-global-trends-2018.html

United Nations High Commissioner for Refugees. (2021). *UNHCR - Global Trends in Forced Displacement – 2020*. https://www.unhcr.org/60b638e37/unhcr-global-trends-2020

Appendix S5: Description of sample from Tanzania

**Background and context:** The Tanzanian sub-sample is part of a cross-sectional epidemiological study investigating associations between exposure to war-related and family violence and psychopathology of Burundian refugee children and their parents. Following Burundi`s independence from the Belgian colonialists in 1962, deeply rooted ethnic tensions between the Hutu and Tutsi groups were misused to gain and secure political power and erupted in several waves of extreme violence, for example in 1972, 1988 and a long-lasting civil war from 1993 until 2005 (Irankunda et al., 2017; Uvin, 2009). It can be assumed that these atrocious traumas in Burundi`s younger history left their marks on the mental health of its citizens, which is supported by studies showing high rates of distress in samples of displaced and non-displaced Burundians (de Jong et al., 2000; Familiar et al., 2016; Yeomans et al., 2008).

In April 2015, Burundi plunged into the latest crisis, when the president Pierre Nkurunziza announced to stay in power for an illegitimate third term. Violence and atrocities committed by members of the ruling party, particularly its youth wing *Imbonerakure*, towards perceived opponents, including abductions, extrajudicial killings and torture, caused more than 400,000 Burundians to flee to neighbouring countries, making Burundi the 10^th^ largest source country for refugees worldwide at the end of 2017 (Human Rights Watch, 2017; UNHCR, 2018b). Tanzania hosted the largest number of Burundian refugees with over 250,000 people as of October 2017, 58% of whom were children (UNHCR, 2018a). The refugees were resettled in three refugee camps, Nyarugusu, Nduta and Mtendeli, in the Kigoma region in Western Tanzania close to the border to Burundi. Nyarugusu camp was opened in 1996 to provide refuge for people fleeing the war in DRC and hosted 69,065 Burundian and 80,080 Congolese refugees as of October 2017 (UNHCR, 2017), making it the third largest refugee camp in the world (International Federation of Red Cross and Red Crescent Societies, 2019). Nduta and Mtendeli camps were reopened in 2015 to relieve the capacities of Nyarugusu and to manage the ongoing influx of refugees, hosting 120,043 and 47,296 Burundian refugees respectively at the end of 2017 (UNHCR, 2017).

**Data collection procedures:** The sample was recruited in the three camps between February and May 2018 in the context of a research project by the Uniersity of Zurich, Switzerland, Bielefeld University, Germany, and the Dar es Salaam University College of Eduaction, Tanzania. A combined systematic and random sampling approach was used to select 230 family triads consisting of the oldest child in primary school age (i.e. between 7 and 15 years), the mother or primary female caregiver and the father or primary male caregiver. Structured clinical interviews were conducted with each family member by Tanzanian psychologists and research assistants recruited from the refugee communities in the camps. Research assistants had been trained in all assessment procedures in a one week training workshop. A detailed description of the study procedures can be found in Scharpf, Kyaruzi, Landolt & Hecker (2019) and Scharpf, Mkinga, Neuner, Machumu & Hecker (2020).

**Measures:** Children were interviewed about their exposure to war-related trauma, maltreatment by parents, mental health problems as well as other aspects of well-being (coping, relationships with parents and peers). All instruments used in the interviews were translated from English to Kiswahili, the lingua franca in the refugee camps, using blind-back translation (Brislin et al., 1973). The instruments had been successfully applied in studies with children in the Great Lakes Region. The contextual applicability of the instruments was further ensured through focus group discussions during the training workshops and a pilot assessment in the first camp Mtendeli.

**Consent and ethics:** The study was approved by the ethics committee of the University of Zurich, National Institute for Medical Research Tanzania (NIMR) and the Commission of Science and Technology in Tanzania (COSTECH). All participants received detailed written and oral information on the study procedures, associated risks, the confidentiality of their data and their rights to terminate their participation at any time without any consequences. All participants provided their informed consent for participation and parents gave their consent on behalf of children below the age of 11. Each family received a compensation of 20 000 Tanzanian Shillings (approximately 8 US Dollars) for their participation. Participants suffering from severe mental health problems were referred to local mental health services within the camps.

**References**

Brislin, R. W., Lonner, W. J., & Thorndike, R. M. (1973). *Cross-cultural research methods: Comparative studies in behavioral science*. John Wiley & Sons.

de Jong, J. T. V. M., Scholte, W. F., Koeter, M. W., & Hart, A. A. (2000). The prevalence of mental health problems in Rwandan and Burundese refugee camps. *Acta Psychiatrica Scandinavica Scand*, *102*(3), 171–177.

Familiar, I., Hall, B., Bundervoet, T., Verwimp, P., & Bass, J. (2016). Exploring Psychological Distress in Burundi During and After the Armed Conflict. *Community Mental Health Journal*, *52*(1), 32–38. https://doi.org/10.1007/s10597-015-9902-4

Human Rights Watch. (2017). *World Report 2017: Burundi*. https://www.hrw.org/world-report/2017/country-chapters/burundi

International Federation of Red Cross and Red Crescent Societies. (2019). *Delivering health and dignity for mothers and babies in the world’s third-largest refugee camp*. https://media.ifrc.org/ifrc/2019/01/14/delivering-health-dignity-mothers-babies-worlds-third-largest-refugee-camp/

Irankunda, P., Heatherington, L., & Fitts, J. (2017). Local terms and understandings of mental health problems in Burundi. *Transcultural Psychiatry*, *54*(1), 66–85. https://doi.org/10.1177/1363461516689004

Scharpf, F., Kyaruzi, E., Landolt, M. A., & Hecker, T. (2019). Prevalence and co-existence of morbidity of posttraumatic stress and functional impairment among Burundian refugee children and their parents. *European Journal of Psychotraumatology*, *10*, 1676005. https://doi.org/10.1080/20008198.2019.1676005

Scharpf, F., Mkinga, G., Neuner, F., Machumu, M., & Hecker, T. (2020). Fuel to the fire: The escalating interplay of attachment and maltreatment in the transgenerational transmission of psychopathology in families living in refugee camps. *Development and Psychopathology*, 1–14. https://doi.org/10.1017/S0954579420000516

UNHCR. (2017). *Tanzania Refugee Situation Statistical Report 31-Oct-17*. https://data2.unhcr.org/en/documents/details/60875

UNHCR. (2018a). *Burundi Situation: Regional Inter Agency Plan of Action for the Protection of Refugee Children*. https://www.unhcr.org/5a683fdf7.pdf

UNHCR. (2018b). *Global Trends: Forced Displacement in 2017.* https://www.unhcr.org/5b27be547.pdf

Uvin, P. (2009). *Life after violence : A people’s story of Burundi*. Zed Books.

Yeomans, P. D., Herbert, J. D., & Forman, E. M. (2008). Symptom comparison across multiple solicitation methods among Burundians with traumatic event histories. *Journal of Traumatic Stress*, *21*(2), 231–234. https://doi.org/10.1002/jts.20325

Appendix S6: Description of sample from Uganda

**Background and context:** The northern Ugandan sub-sample is part of a longitudinal epidemiological study investigating the relationship between war experience, family violence and parental and child psychopathology as well as long-term development in adolescence. The subsample used here consists of children who were particularly affected by the violent conflict between the Ugandan government and the Lord Resistance Army (LRA) from 1986-2006. The war had a major impact on civilians in the Acholi region of northern Uganda. Military raids and abductions by the LRA and government forces led to the forced internment of nearly 90 % of the population in camps for internally displaced people (IDP; Bjorkhaug, Morten, Hatloy, & Jennings, 2007; International Crisis Group, 2006). During their internment families experienced high levels of psychopathology as well as high levels of intimate partner violence, violent parenting and the erosion of social relationships (Ertl, Pfeiffer, Schauer-Kaiser, Elbert, & Neuner, 2014; Hovil & Moorhead, 2002; Roberts, Ocaka, Browne, Oyok, & Sondorp, 2008; Uganda Bureau of Statistics & Macro International, 2007). Since 2007 families started to return to their communities of origin.

**Data collection procedures:** In 2010, 368 second graders and their primary guardians living in nine war-affected communities near former IDP camps were recruited. The communities were purposively sampled and all second-grader and their primary guardians in the selected communities were included. To address the varying degrees of literacy in the sample, all instruments were administered as structured interviews in Luo Acholi by trained local counselors (5 male, 9 female; clinical and research experience between 2-11 years, *M* = 7.14) under the supervision of an international team of clinical experts with extensive experience in transcultural research. A detailed description of the sample recruitment and the instruments used can be found in Saile, Ertl, Neuner, & Catani (2014, 2016) and Saupe, Gößmann, Catani, & Neuner 2019, 2020), among others.

**Measures:** Children were interviewed about their exposure to war-related trauma, family violence, various mental health problems as well as psychological and social resources. All instruments used in the interviews were translated into Luo Acholi, the local language, following recommended procedures in transcultural research (Flaherty et al., 1988; van Ommeren et al., 1999) including translation, lexical back translation, blind back translation, and separate focus group discussions with bilingual local mental health counselors as well as study participants. All theoretical concepts and measures were discussed with a panel of local counselors and international clinical experts to ensure the adequacy of the questions.

**Consent and ethics:** The study protocol was approved by the ethics committee of the German Research Foundation (DFG), the ethics committee of Gulu University in Uganda, and the National Council for Science and Technology in Uganda (UNCST). At each wave of data collection, all participating children and their primary guardians were invited to an information meeting where the study objectives and procedures were explained. In the case of the children, written consent by at least one guardian as well as by the child was obtained. No monetary incentives were given, but all participants received a snack during the interview. In cases of severe psychopathological outcomes, participants were referred to local counselors who provided treatment.

**References**

Saile, R., Ertl, V., Neuner, F., & Catani, C. (2014). Does war contribute to family violence against children? Findings from a two-generationalratinal multi-informant study in Northern Uganda. *Child Abuse & Neglect*, (1), 135–146.

Saile, R., Ertl, V., Neuner, F., & Catani, C. (2016). Children of the postwar years: A two-generational multilevel risk assessment of child psychopathology in northern Uganda. *Development and Psychopathology*, *28*(2), 607–620. https://doi.org/10.1017/S0954579415001066

Saupe, L. B., Gößmann, K., Catani, C., & Neuner, F. (2019). Adolescent Life Perspectives After War: Evaluation and Adaptation of the Future Expectation Scale in Uganda. *Frontiers in Psychology*, *10*(July), 1–14. https://doi.org/10.3389/fpsyg.2019.01527

Saupe, L. B., Gößmann, K., Catani, C., & Neuner, F. (2020). Child Abuse & Neglect Understanding the link between child maltreatment and adolescent future expectations in Northern Uganda : A serial mediation analysis. *Child Abuse & Neglect*, *106*(April), 104511. https://doi.org/10.1016/j.chiabu.2020.104511

**Appendix S7:** R script for the analyses

*#Network analysis with total sample*

*#Loading required packages*

library(bootnet)

library(qgraph)

library(foreign)

library(mgm)

library(NetworkComparisonTest)

*#Load the dataset*

network.data<-read.spss("name.sav", use.value.labels=FALSE, to.data.frame=TRUE)

*#Estimate the network*

Network.total <- estimateNetwork(network.data,default = "EBICglasso")

*#Plot the network*

plot(Network.total, layout = "spring")

*#Accuracy and stability analyses*

*#Computing edge-weight accuracy*

boot1 <- bootnet(Network.total, nBoots = 2500, nCores = 8)

*#Display of bootstrapped CIs for estimated edge parameters*

plot(boot1, labels = FALSE, order = "sample")

*#Investigate stability of centrality indices*

boot2 <- bootnet(Network.total, nBoots = 2500, type = "case", nCores = 8, statistics =c("edge", "betweenness", "closeness", "strength", "expectedInfluence"))

*#Plot stability of centrality indices*

plot(boot2, "strength")

plot(boot2, "expectedInfluence")

plot(boot2, "closeness")

plot(boot2, "betweenness")

**Appendix S7:** R script for the analyses (continued)

*#Calculation of stability coefficients*

corStability(boot2)

*#Plot centrality indices*

centralityPlot(Network.total, include = c("Strength", "ExpectedInfluence", "Closeness"))

*#Plot differences in centrality indices between nodes*

plot(boot1, "strength")

plot(boot1, "expectedInfluence")

plot(boot1, "closeness")

*#Network analyes based on age*

*#Children`s network*

*#Load the dataset*

network.data.children <-read.spss("name.sav", use.value.labels=FALSE, to.data.frame=TRUE)

*#Estimate the network*

Network.children <- estimateNetwork(network.data.young,default = "EBICglasso")

*#Plot the network*

plot(Network.children, layout = "spring")

*#Accuracy and stability analyses*

*#Computing edge-weight accuracy*

boot3 <- bootnet(Network.children, nBoots = 2500, nCores = 8)

*#Display of bootstrapped CIs for estimated edge parameters*

plot(boot3, labels = FALSE, order = "sample")

*#Investigate stability of centrality indices*

boot4 <- bootnet(Network.children, nBoots = 2500, type = "case", nCores = 8, statistics =c("edge", "betweenness", "closeness", "strength", "expectedInfluence"))

**Appendix S7:** R script for the analyses (continued)

*#Plot stability of centrality indices*

plot(boot4, "strength")

plot(boot4, "expectedInfluence")

plot(boot4, "closeness")

plot(boot4, "betweenness")

*#Calculation of stability coefficients*

corStability(boot4)

*#Plot centrality indices*

centralityPlot(Network.children, include = c("Strength", "ExpectedInfluence"))

*#Plot differences in centrality indices between nodes*

plot(boot3, "strength")

plot(boot3, "expectedInfluence")

*#Estimate predictability of nodes*

type=rep('g', p)

pred.children <- mgm(data = network.data.children, type = rep('g', 17), level = rep(1, 17), lambdaSel = 'CV', ruleReg = 'OR', pbar = FALSE)

pred_obj_children <- predict(object = pred.children, data = network.data.children, errorCon = 'R2')

pred_obj_children$error

*#Adolescents` network*

*#Load SPSS dataset*

network.data.adolescents <-read.spss("/Users/fscharpf/Desktop/Promotion/Schreibprozess/PTSD network paper youth/17symptoms_13_18_1.sav", use.value.labels=FALSE, to.data.frame=TRUE)

*#Estimate the network*

Network.adolescents <- estimateNetwork(network.data.adolescents,default = "EBICglasso")

**Appendix S7:** R script for the analyses (continued)

*#Plot the network*

plot(Network.adolescents, layout = "spring")

*#Accuracy and stability analyses*

*#Computing edge-weight accuracy*

boot5 <- bootnet(Network.adolescents, nBoots = 2500, nCores = 8)

*#Display of bootstrapped CIs for estimated edge parameters*

plot(boot5, labels = FALSE, order = "sample")

*#Investigate stability of centrality indices*

boot6 <- bootnet(Network.adolescents, nBoots = 2500, type = "case", nCores = 8, statistics =c("edge", "betweenness", "closeness", "strength", "expectedInfluence"))

*#Plot stability of centrality indices*

plot(boot6, "strength")

plot(boot6, "expectedInfluence")

plot(boot6, "closeness")

plot(boot6, "betweenness")

*#Calculation of stability coefficients*

corStability(boot6)

*#Plot centrality indices*

centralityPlot(Network.adolescents, include = c("Strength", "ExpectedInfluence"))

*#Plot differences in centrality indices between nodes*

plot(boot5, "strength")

plot(boot5, "expectedInfluence")

*#Estimate predictability of nodes*

type=rep('g', p)

pred.adolescents <- mgm(data = network.data.adolescents, type = rep('g', 17), level = rep(1, 17), lambdaSel = 'CV', ruleReg = 'OR', pbar = FALSE)

**Appendix S7:** R script for the analyses (continued)

pred_obj_adolescents <- predict(object = pred.adolescents, data = network.data.adolescents, errorCon = 'R2')

pred_obj_adolescents$error

*#Plot the children and adolescents‘ network with identical layout*

L <- averageLayout(Network.children, Network.adolescents, layout = "spring")

plot(Network.children, layout = L)

plot(Network.adolescents, layout = L)

*#Plot centrality indices for the children and adolescents‘ network in one figure*

age.list <- list(Children = Network.children, Adolescents = Network.adolescents)

centralityPlot(age.list, include = c("Strength", "ExpectedInfluence"))

*#Comparing the children and adolescents‘ network*

age.differences <- NCT(network.data.children, network.data.adolescents, gamma=0.25, it=200, binary.data=FALSE, paired=FALSE, weighted=TRUE,AND=TRUE, test.edges=TRUE, edges="all", progressbar=TRUE, make.positive.definite=TRUE, p.adjust.methods= c("none"), test.centrality=TRUE,centrality=c("strength","expectedInfluence"), nodes="all",communities=NULL,useCommunities="all",verbose = TRUE)

*#Obtaining summary statistics*

age.differences

|  | Complete sample  *N* =2007 | Gaza  *n* = 482 | DRC  *n* = 252 | Tanzania  *n* = 230 | Uganda  *n* = 368 | Iraq  *n* = 332 | Burundi  *n* = 343 | Group comparisons (sub-samples)^3^ |
| --- | --- | --- | --- | --- | --- | --- | --- | --- |
| Age  mean, (SD) | 11.92 (2.70) | 11.29  (.68) | 15.73  (1.66) | 12.11  (2.03) ^cf^ | 9.01  (1.25) | 12.67  (2.08) ^ef^ | 12.28  (3.22)^cef^ | F (5;2001) = 389,23 ***, r =.70 |
| Gender (female),  n (%)^4^ | 999 (49.78) | 240 (49.79)  χ_avsb_^2^ (1) = 9.14*  χ_avsc_^2^ (1) = .36 ^ns^  χ_avsd_^2^ (1) = 1.43  ^ns^  χ_avse_^2^ (1) = .32 ^ns^  χ_avsf_^2^ (1) = 1.70  ^ns^ | 155 (61.51)  χ_bvsc_^2^ (1) = 9.67*  χ_bvsd_^2^ (1) = 15.07***  χ_bvse_^2^ (1) = 5.47 ^ns^  χ_bvsf_^2^ (1) = 15.50*** | 109 (47.39)  χ_cvsd_^2^ (1) = .74  ^ns^  χ_cvse_^2^ (1) = .35 ^ns^  χ_cvsf_^2^ (1) = .61  ^ns^ | 168 (46.65)  χ_dvse_^2^ (1) = 2.65 ^ns^  χ_dvsf_^2^ (1) = .02  ^ns^ | 172 (51.81)  χ_fvse_^2^ (1) = 2.96 ^ns^ | 155 (45.19) |  |
| Trauma category load^1^  mean, (SD) | 3.07 (1.56) | 2.31  (1.08) | 4.81 (1.39) | 3.37  (1.53) ^ce^ | 2.97 (1.63)^def^ | 3.11 (1.43)^cde^ | 2.75 (1.28)^df^ | F (5;2001) = 116,13 ***, r =.47 |
| PTSD severity^2^ mean, (SD) | 8.97 (11.43) |  |  | 12.42 (10.13)^cef^ | 2.73  (3.90) | 12.24 (16.24)^cef^ | 10.16 (9.15)^cef^ | F (3;1268) = 114,33 ***, r =.36 |

**Table S1.** Descriptive information and frequencies for the complete sample and the sub-samples as well as group comparisons across sub-samples.

*Note.*^1^Trauma category load reflects the sum of different traumatic event categories experienced by each child. ^2^ PTSD severity was calculated by summing up PTSD scores for all 17 PTSD symptoms according to the DSM IV. As the Gaza and the DRC sub-sample did not provide information on all 17 PTSD symptoms, PTSD severity was only calculated for the remaining sub-sample sets. ^3^ Continuous variables were compared across sub-samples using one-way ANOVA with Hochbergs’ GT2 post-hoc test to account for the differences in sub-sample size. Superscripts (^abcd^) indicate significant class differences (Gaza =^a^, DRC =^b^, Tanzania =^c^, Uganda = ^d^, Iraq = ^e.^, Burundi = ^f^). ^4^ Dichotomous variables were compared across sub-samples using Chi ^2^ Statistics, with Bonferroni-Holm corrected p-values. *= p<.05, **p<.005,*** p<.001, ns=non-significant

|  | Complete sample  *N* =885 | Children (6-12 years)  *n* = 412 | Adolescents (13-18 years)  *n* = 473 | Group comparisons (age groups)^3^ |
| --- | --- | --- | --- | --- |
| Age  mean, (SD) | 12.27 (2.70) | 9,83 (1.62) | 14.40 (1.27) |  |
| Gender (female),  n (%)^4^ | 408 (46.10) | 189 (45.87) | 219 (46.30) | χ_avsb_^2^ (1) = .016^ns^ |
| Trauma category load^1^  mean, (SD) | 3.36 (1.43) | 3.43 (1.48) | 3.29 (1.39) | F (1;883) = 1.42 ^ns^ |
| PTSD severity^2^ mean, (SD) | 12.69 (11.93) | 12.18 (10.27) | 13.14 (13.19) | F (1;883) = .1.84 ^ns^ |

**Table S2.** Descriptive information and frequencies for the sub-sample used for the comparison of networks for age groups.

*Note.*^1^Trauma category load reflects the sum of different traumatic event categories experienced by each child. ^2^ PTSD severity was calculated by summing up PTSD scores for all 17 PTSD symptoms according to the DSM IV. ^3^ Continuous variables were compared across age group sub-samples using one-way ANOVA with Hochbergs’ GT2 post-hoc test to account for the differences in sub-sample size. Superscripts (^abcd^) indicate significant class differences (Children =^a^, Adolescents =^b^). ^4^ Dichotomous variables were compared across sub-samples using Chi ^2^ Statistics, *= p<.05, **p<.005,*** p<.001, ns=non-significant

Table S3. Descriptive statistics of PTSD symptoms in the overall sample and in the sub-samples based on age

|  | **Overall sample (n = 2007)** | | | | | **Sub-sample for age comparisons (n = 885)** | | | | | |
| --- | --- | --- | --- | --- | --- | --- | --- | --- | --- | --- | --- |
|  |  | | | | | **Children aged 6 – 12 (n = 412)** | | | **Adolescents aged 13 -18 (n = 473)** | | |
|  |  | | | | |  | | |  | | |
|  | n | % | M | SD | Skewness | M | SD | Skewness | M | SD | Skewness |
|  |  |  |  |  |  |  |  |  |  |  |  |
| Intrusive memories | 1755 | 87.4 | 1.04 | 1.26 | 0.80 | 0.84 | 1.17 | 1.37 | 0.91 | 1.30 | 1.22 |
| Distressing dreams | 1525 | 76.0 | 0.90 | 1.18 | 1.26 | 1.22 | 1.10 | 0.85 | 0.93 | 1.20 | 1.14 |
| Dissociative reactions | 1525 | 76.0 | 0.58 | 1.04 | 1.86 | 0.58 | 0.99 | 1.82 | 0.73 | 1.07 | 1.43 |
| Psychological distress | 1755 | 87.4 | 1.05 | 1.20 | 0.81 | 1.12 | 1.20 | 0.90 | 1.17 | 1.28 | 0.82 |
| Physiological reactions | 1273 | 63.4 | 0.59 | 1.03 | 1.82 | 0.82 | 1.13 | 1.35 | 0.86 | 1.17 | 1.22 |
| Avoidance of internal reminders | 2007 | 100 | 1.02 | 1.28 | 0.87 | 0.73 | 1.14 | 1.54 | 0.93 | 1.29 | 1.16 |
| Avoidance of external reminders | 2007 | 100 | 1.13 | 1.27 | 0.69 | 0.89 | 1.19 | 1.31 | 1.04 | 1.25 | 0.96 |
| Amnesia | 1273 | 63.4 | 0.25 | 0.78 | 3.50 | 0.26 | 0.78 | 3.37 | 0.44 | 1.00 | 2.41 |
| Exaggerated negative cognitions | 1273 | 63.4 | 0.58 | 0.99 | 1.69 | 0.74 | 1.01 | 1.30 | 0.90 | 1.16 | 1.06 |
| Distorted blaming | 905 | 45.1 | 0.44 | 0.94 | 2.28 |  |  |  |  |  |  |
| Persistent negative emotions | 905 | 45.1 | 1.05 | 1.24 | 0.89 |  |  |  |  |  |  |
| Diminished interest | 1273 | 63.4 | 0.35 | 0.89 | 2.82 | 0.50 | 1.01 | 2.19 | 0.51 | 1.05 | 2.18 |
| Detachment | 1273 | 63.4 | 0.37 | 0.89 | 2.69 | 0.42 | 0.90 | 2.48 | 0.63 | 1.11 | 1.79 |
| Inability to experience positive emotions | 1273 | 63.4 | 0.31 | 0.80 | 3.00 | 0.38 | 0.85 | 2.52 | 0.50 | 0.98 | 2.22 |
| Irritable or aggressive behavior | 1755 | 87.4 | 0.78 | 1.06 | 1.18 | 0.84 | 1.01 | 1.25 | 0.83 | 1.12 | 1.29 |
| Reckless or self-destructive behavior | 905 | 45.1 | 0.10 | 0.43 | 5.33 |  |  |  |  |  |  |
| Hypervigilance | 2007 | 100 | 0.83 | 1.13 | 1.16 | 0.79 | 1.03 | 1.41 | 0.71 | 1.08 | 1.45 |
| Exaggerated startle | 2007 | 100 | 0.91 | 1.17 | 1.04 | 0.82 | 1.04 | 1.32 | 0.80 | 1.18 | 1.41 |
| Concentration problems | 1755 | 87.4 | 0.66 | 1.00 | 1.33 | 0.54 | 0.93 | 1.87 | 0.53 | 0.91 | 1.88 |
| Sleeping difficulties | 1755 | 87.4 | 0.70 | 1.06 | 1.40 | 0.65 | 1.04 | 1.58 | 0.73 | 1.14 | 1.59 |

*Figure S1*. Density plots of PTSD symptoms in children and adolescents

*Figure S1*. Density plots of PTSD symptoms in children and adolescents (continued)


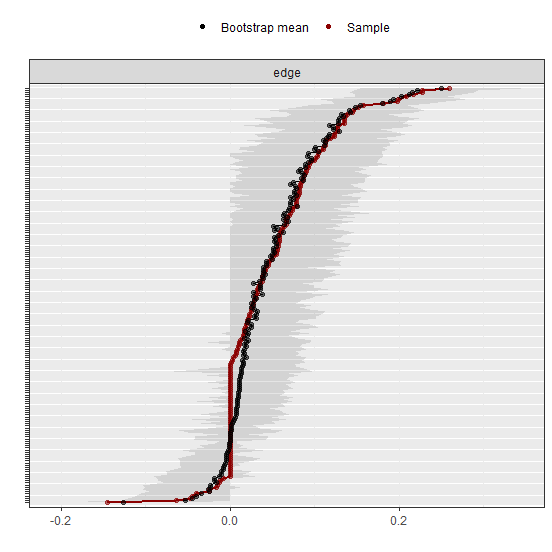


*Figure S2*. Bootstrapped confidence intervals around the edge weights in the network of the overall sample


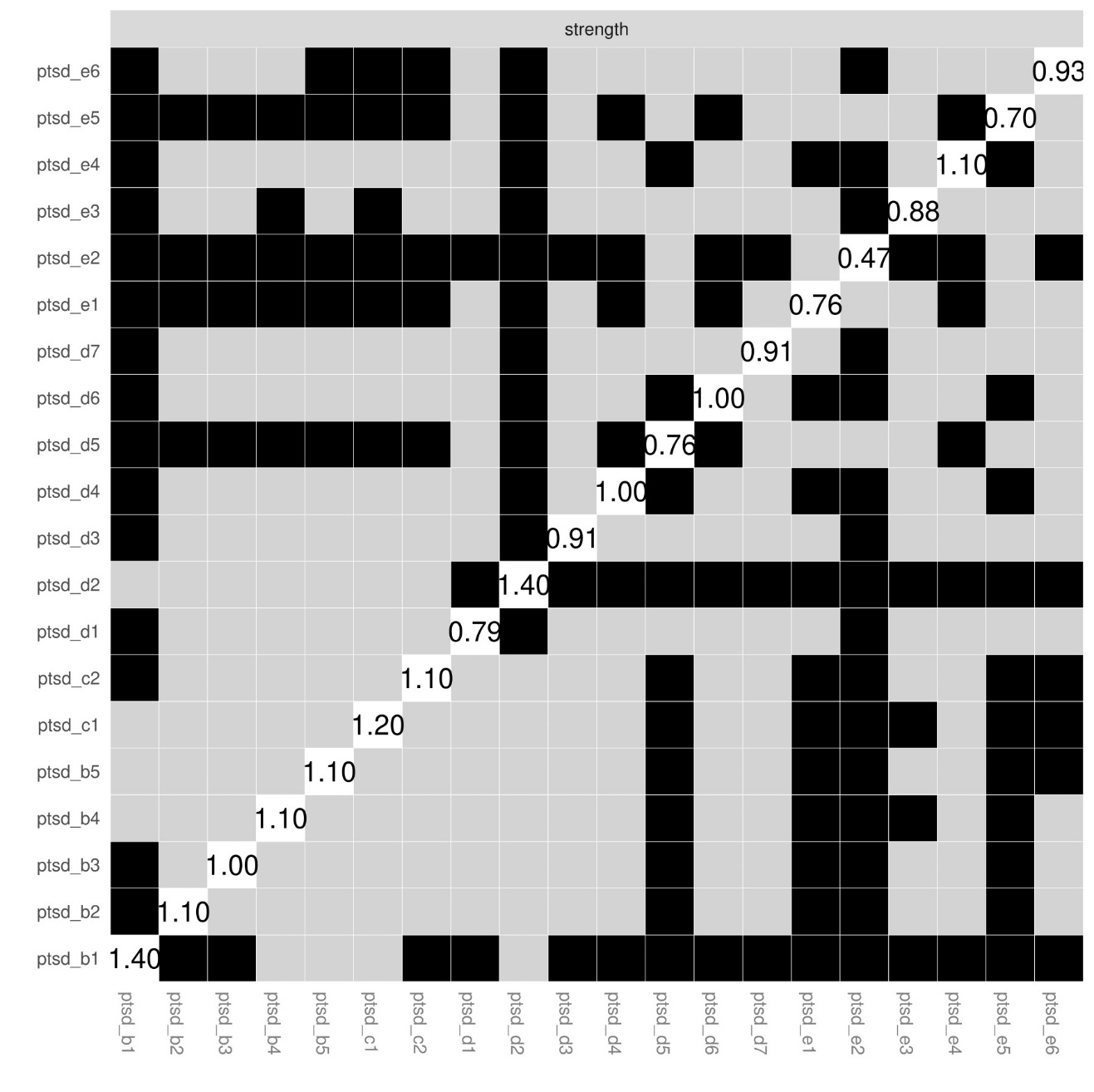


*Figure S3*. Differences between symptoms in node strength in the network of the overall sample


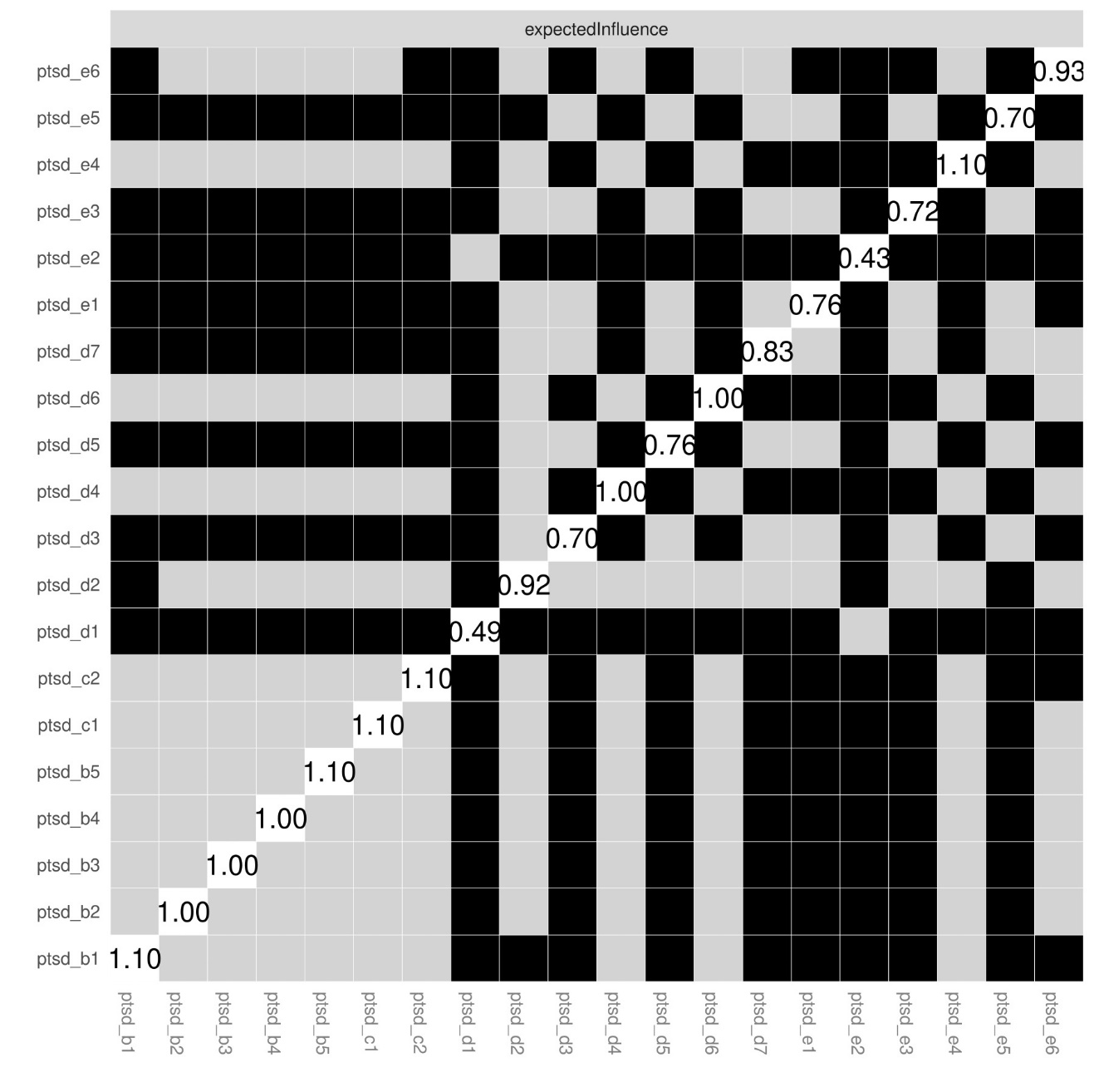


*Figure S4*. Differences between symptoms in expected influence in the network of the overall sample


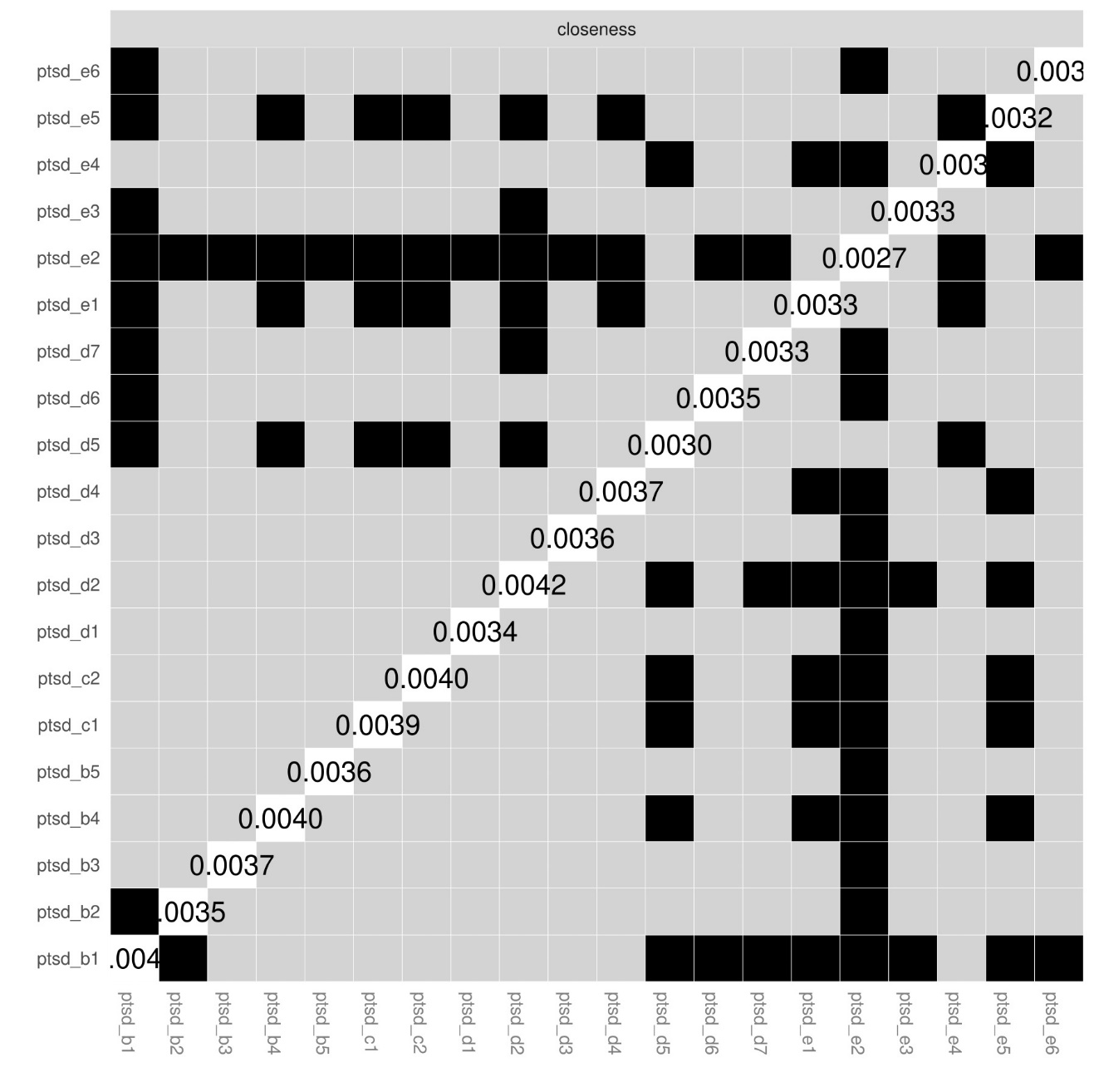


*Figure S5*. Differences between symptoms in closeness in the network of the overall sample


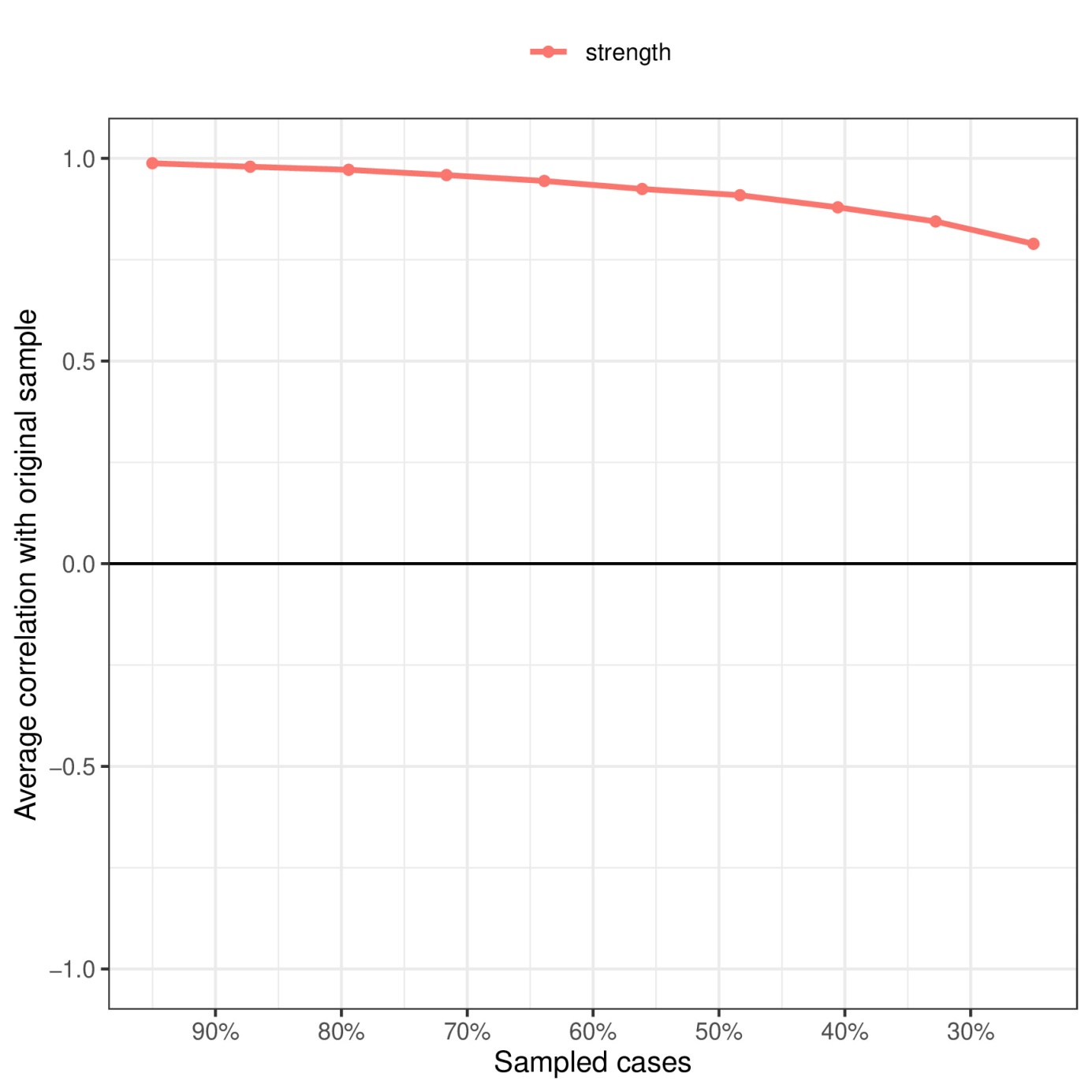


*Figure S6.* Stability of node strength in the network of the overall sample (CS-coefficient: 0.67)


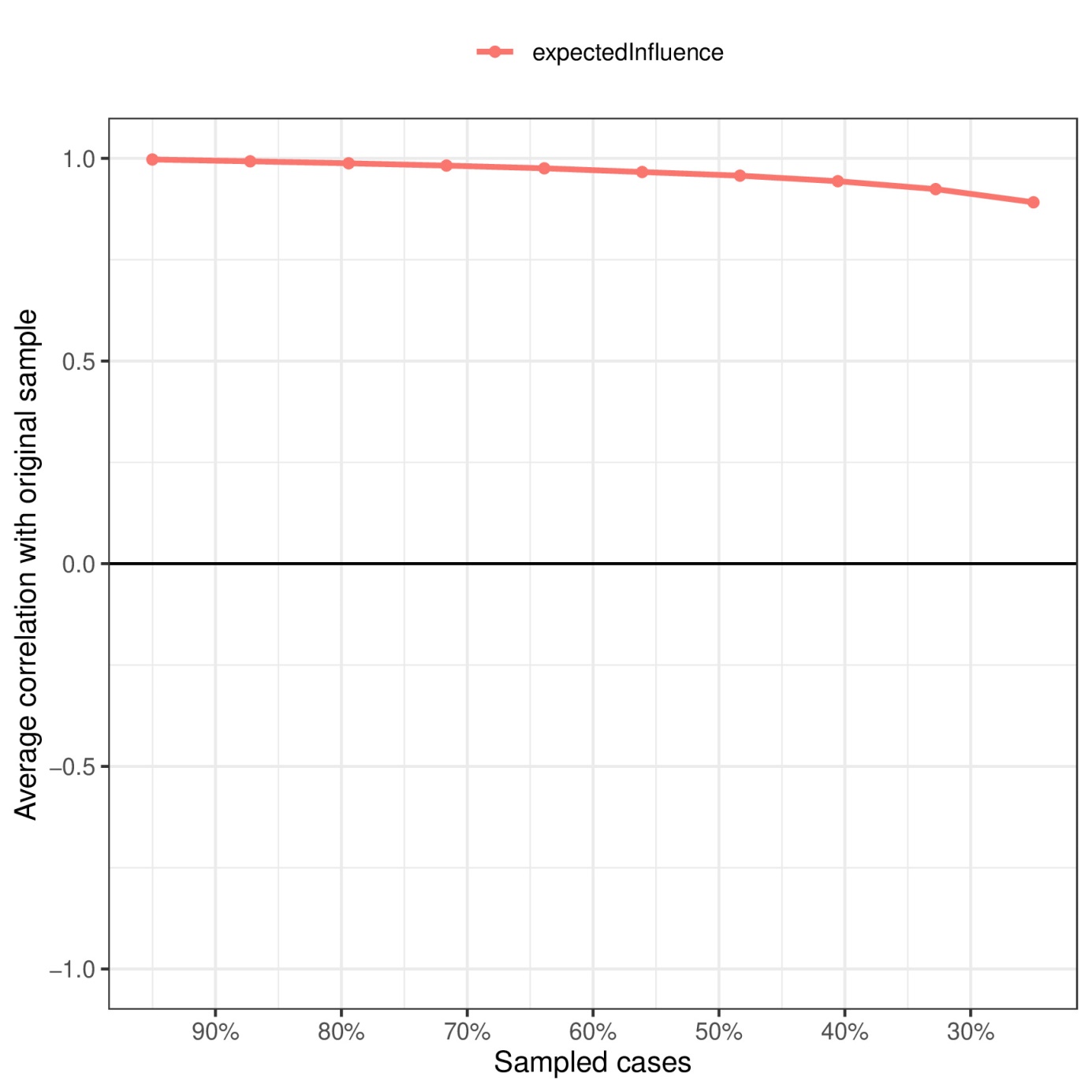


*Figure S7*. Stability of expected influence in the network of the overall sample (CS-coefficient: 0.75)


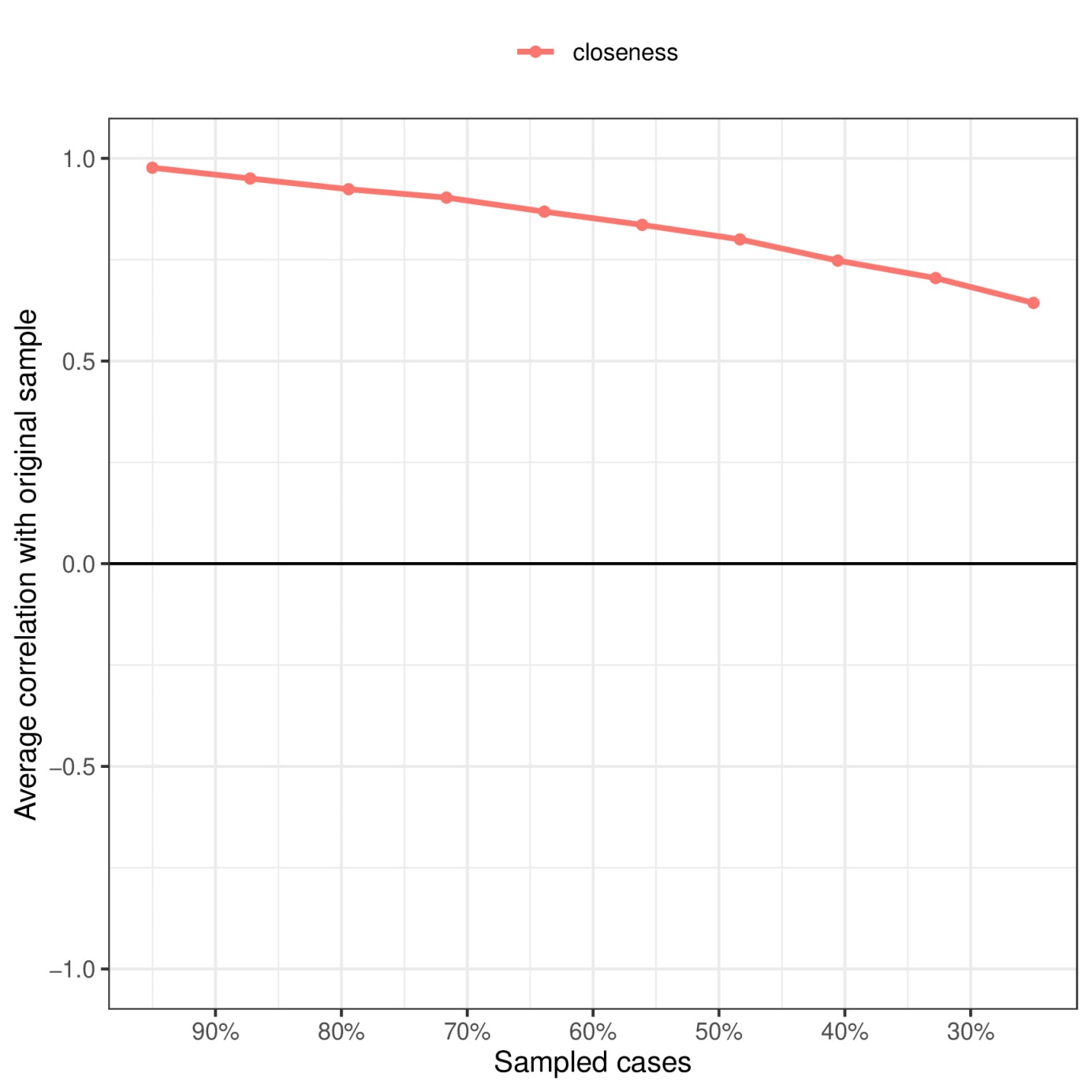


*Figure S8*. Stability of closeness in the network of the overall sample (CS-coefficient: 0.44)


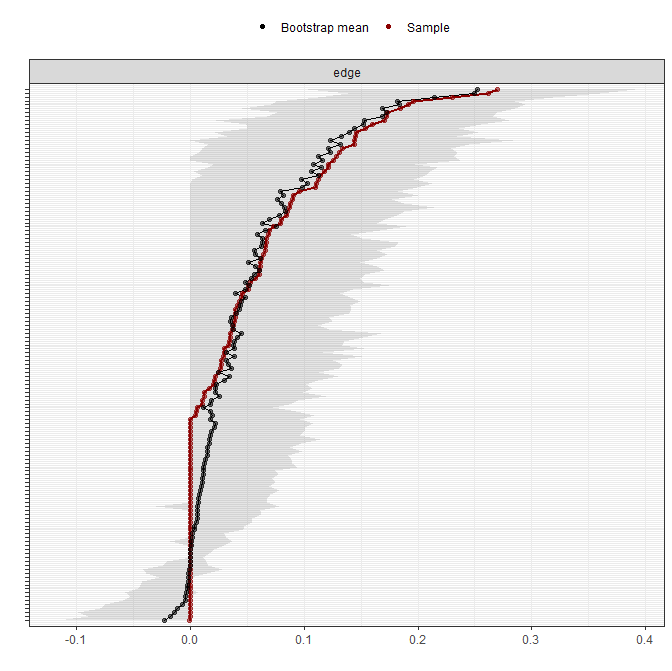

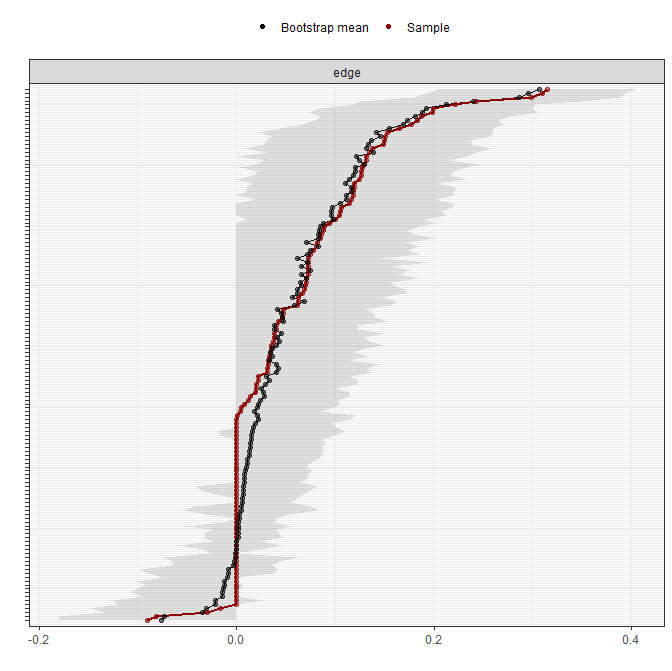


*Figure S9*. Bootstrapped confidence intervals around the edge weights in the networks of children (left) and adolescents (right)


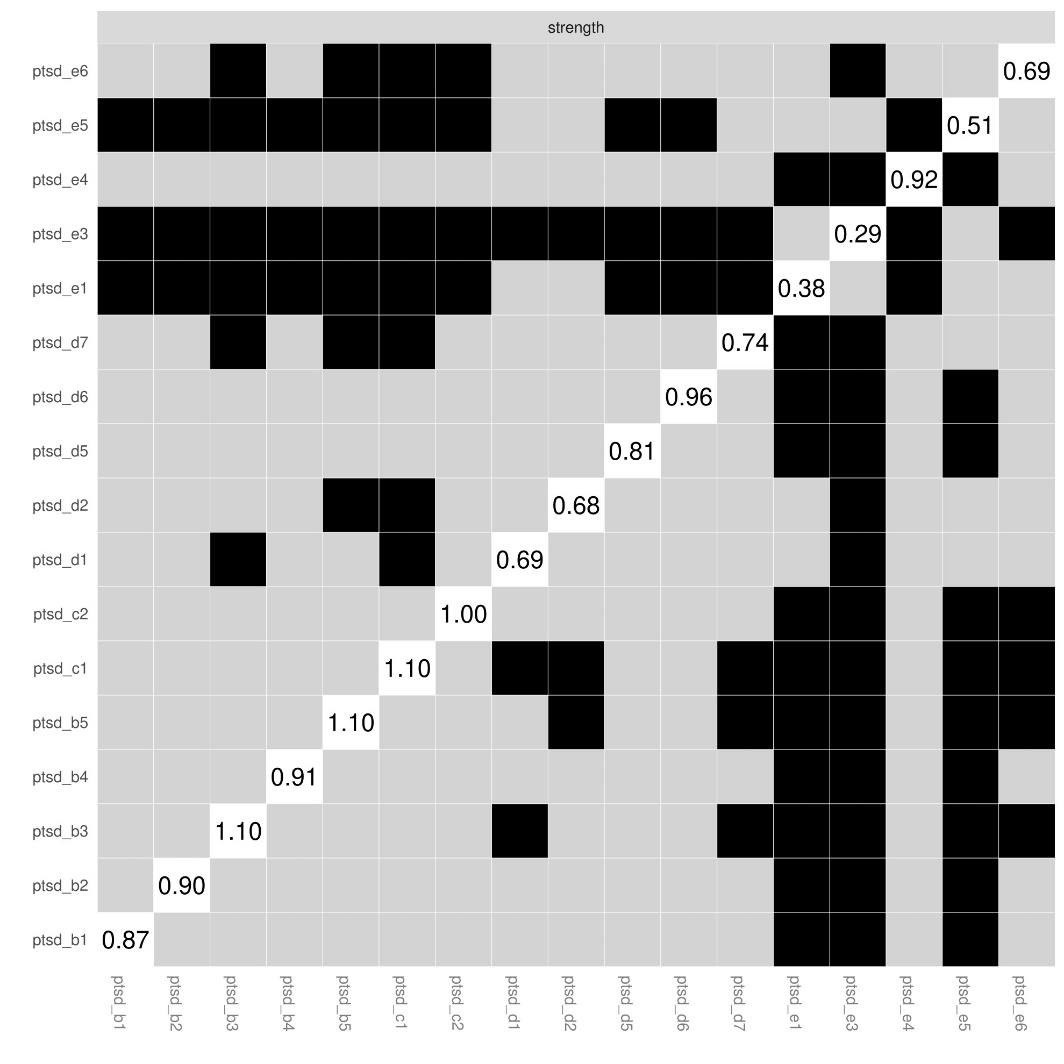

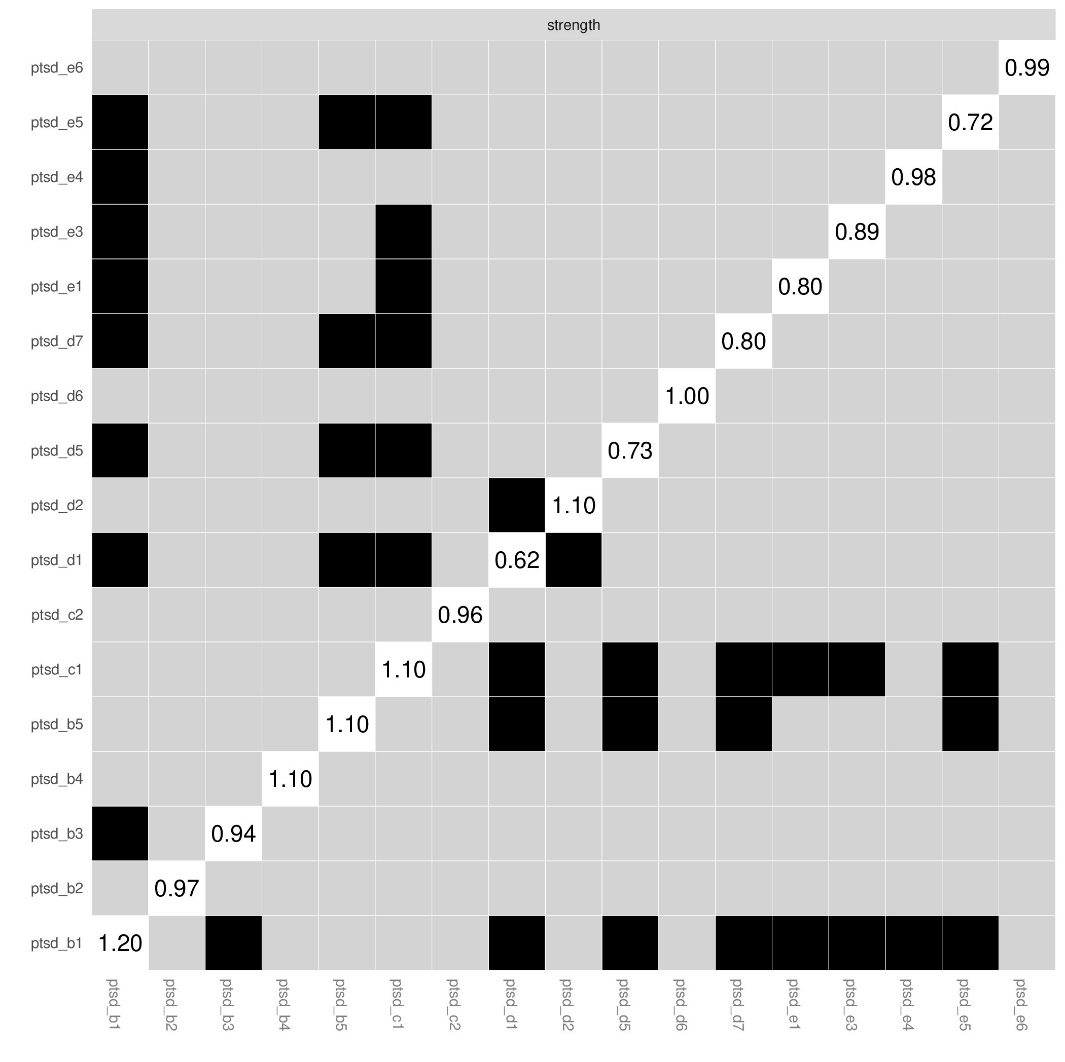


*Figure S10*: Differences between symptoms in node strength in the network of children (left) and adolescents (right)


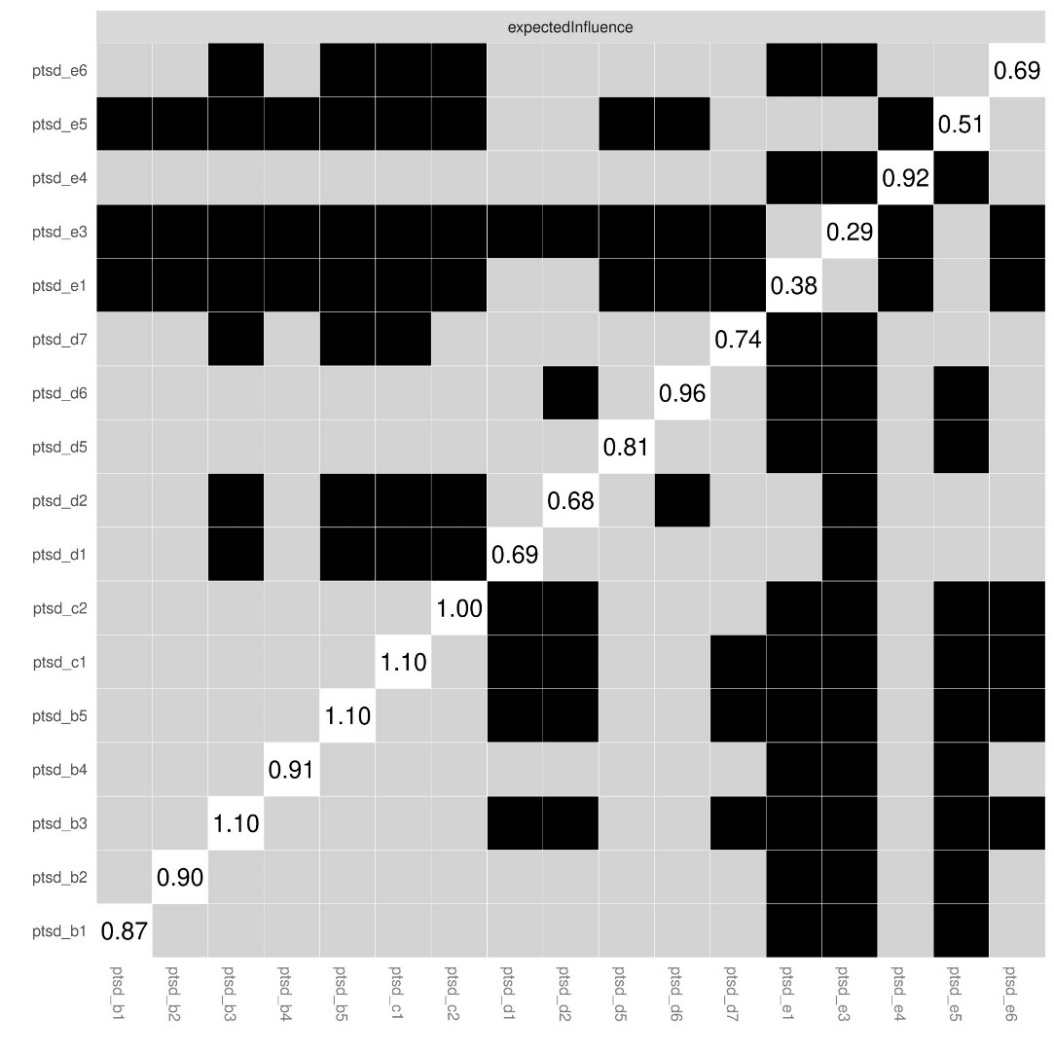

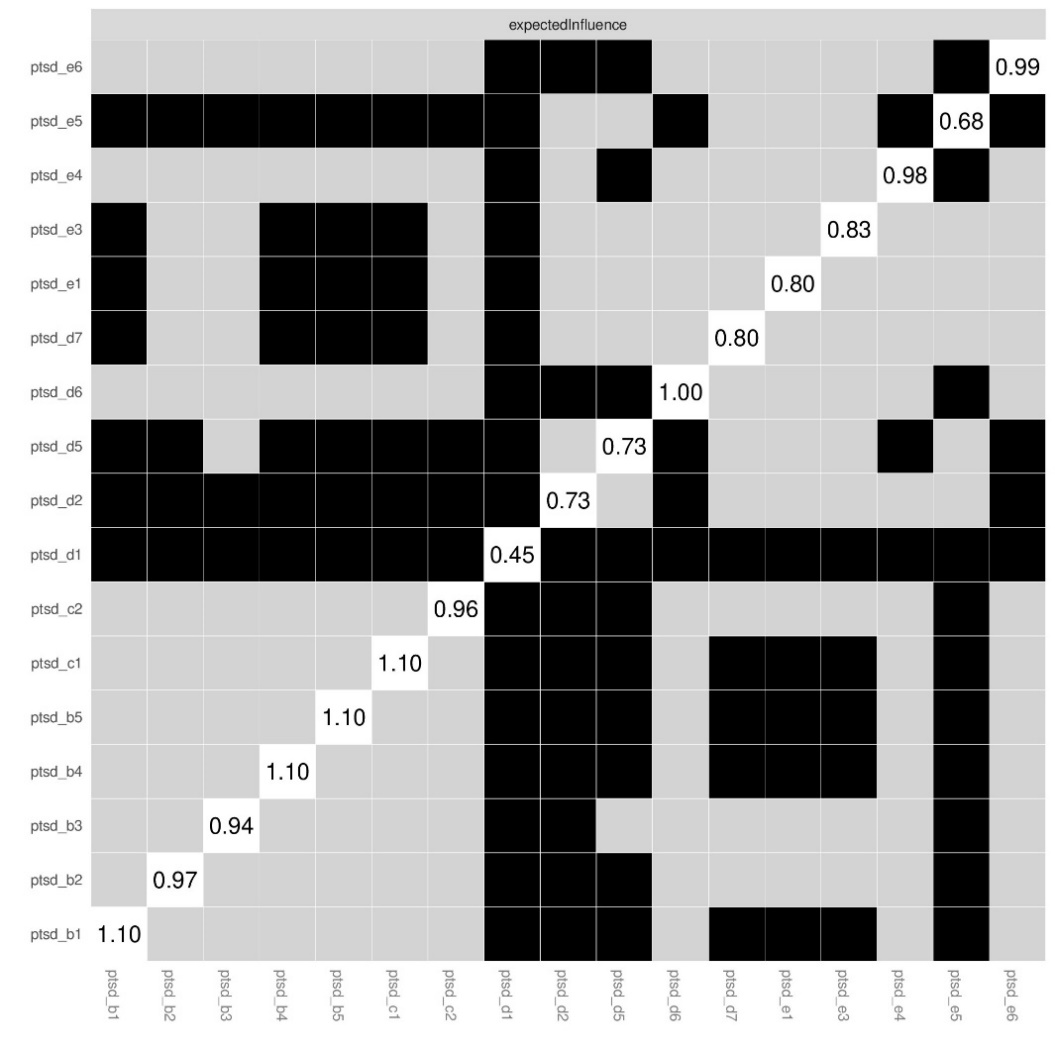


*Figure S11*. Differences in expected influence in the network of children (left) and adolescents (right)


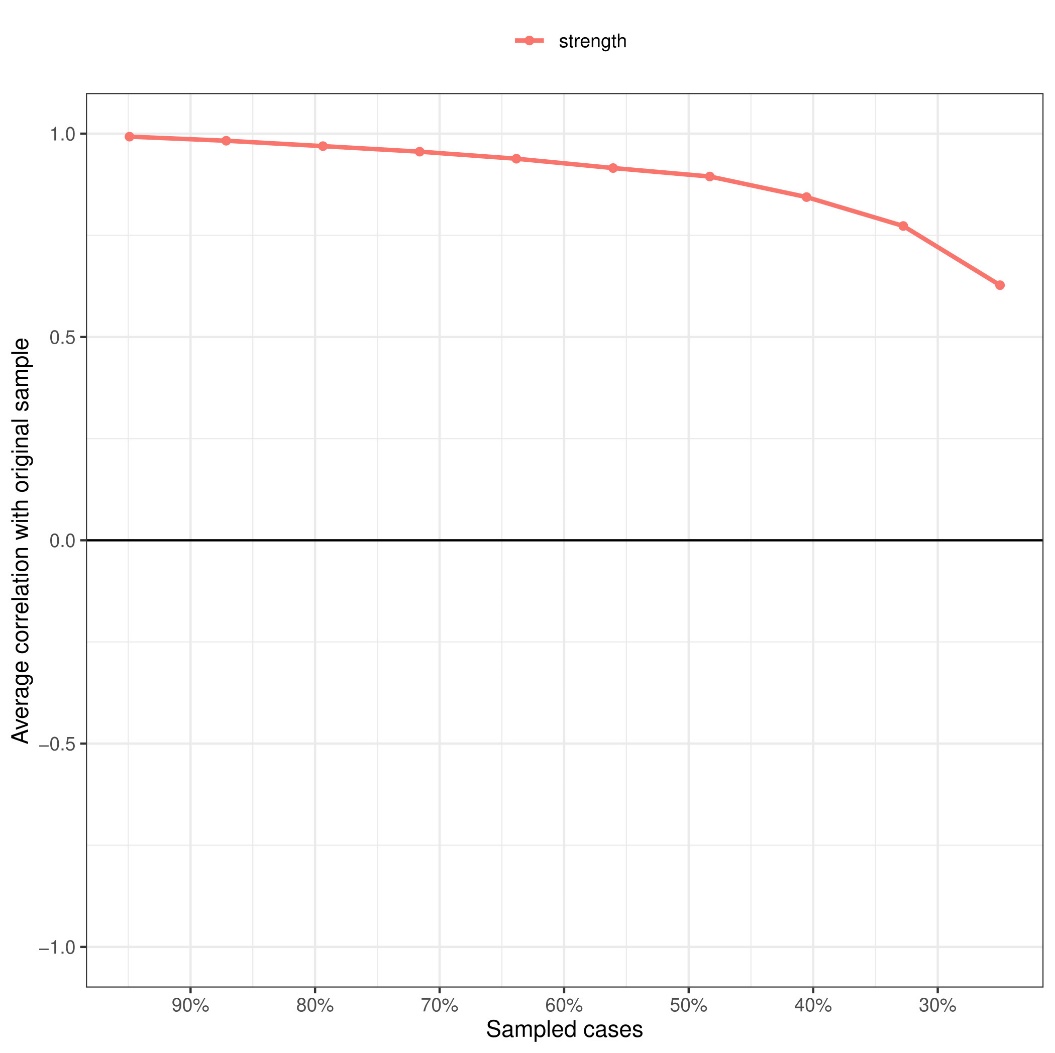

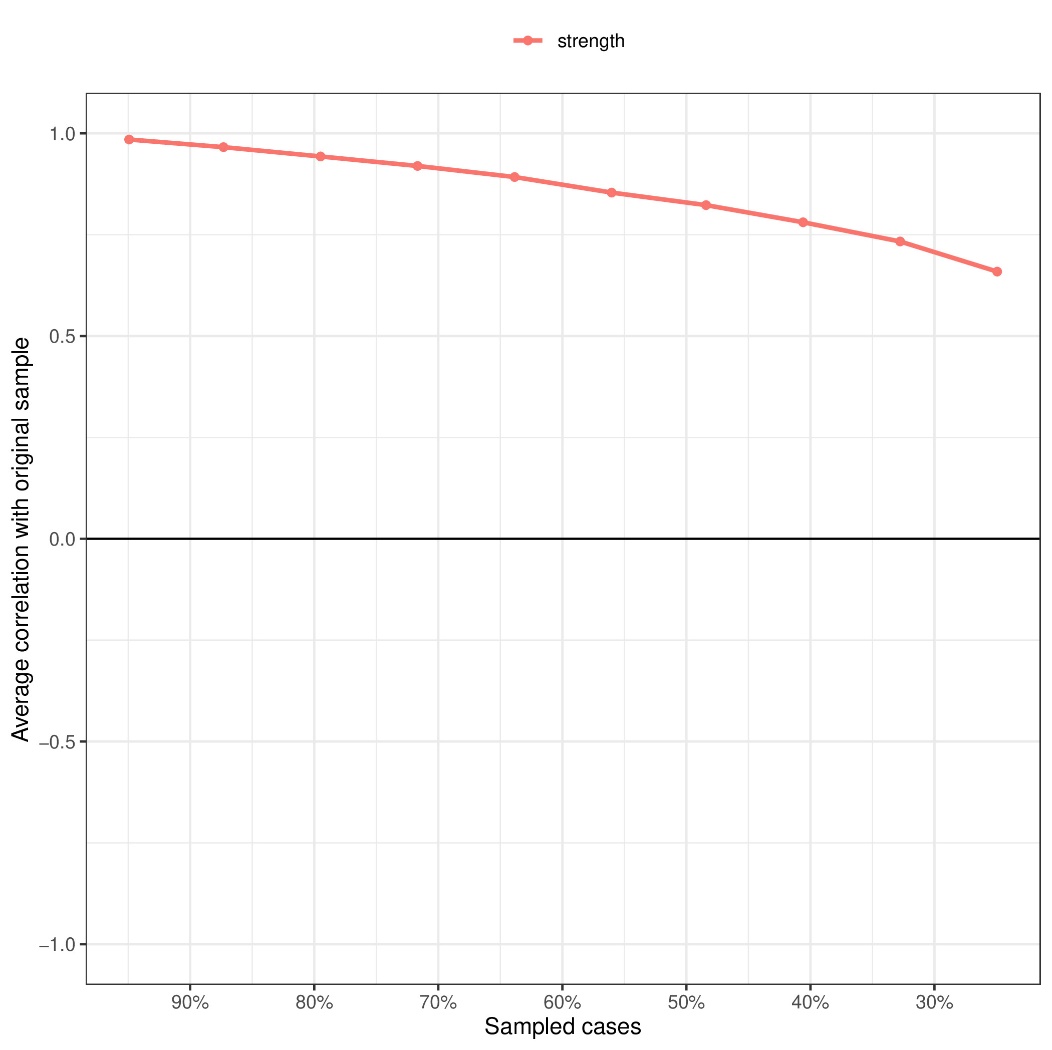


*Figure S12*. Stability of node strength in the network of children (left; CS-coefficient: 0.52) and adolescents (right; CS-coefficient: 0.44)


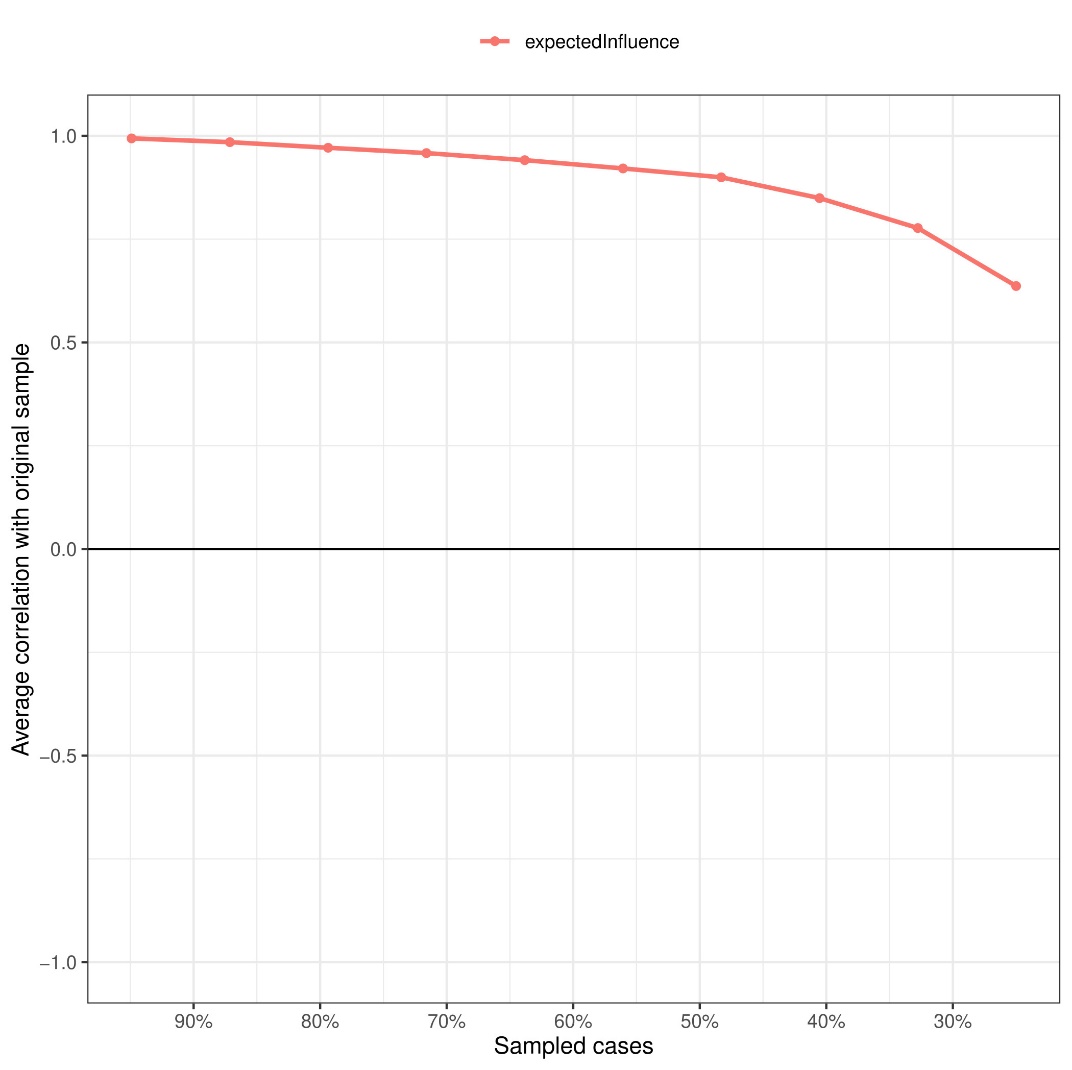

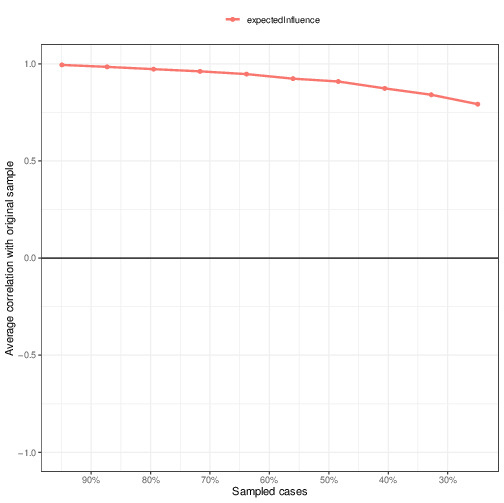


*Figure S13*. Stability of expected influence in the network of children (left; CS-coefficient: 0.52) and adolescents (right; CS-coefficient: 0.67)

1. Le Bureau pour le Volontariat au service de l'Enfance et de la Santé (BVES), Centre d'Apprentissage Professionnel et Artisanal (CAPA), Collectif des Ongs Unies pour le Developpement Durable des Associations Pour L’encadrement Des Personnes Deœuvrees et Vulnerables COUD / AEPDV (Grand Lac), Centre ESSOLE, Association de Développement, Lutte contre la Pauvreté Et pour la Défense des Droits de la Femme (ADPF), Hekabana, and Laissez l'Afrique Vivre (LAV). [↑](#footnote-ref-1)
